# Genome-wide identification of modulators of *Chlamydia trachomatis* parasitophorous vacuole stability highlights an important role for sphingolipid supply

**DOI:** 10.1371/journal.pbio.3003297

**Published:** 2025-08-12

**Authors:** Mohammed Rizwan Babu Sait, Lana H. Jachmann, Gözde Türköz, Milica Milivojevic, Celia Llorente-Sáez, Soniya Dhanjal, Fabian Schumacher, Sara Henriksson, Naga Venkata Gayathri Vegesna, Noha Seddik, Anastasiia Chaban, Partha Mohanty, Magnus Ölander, Samada Muraleedharan, Sepideh Farmand Azadeh, Burkhard Kleuser, Bernhard Schmierer, Barbara S. Sixt

**Affiliations:** 1 Department of Molecular Biology, Umeå University, Umeå, Sweden; 2 The Laboratory for Molecular Infection Medicine Sweden (MIMS), Umeå University, Umeå, Sweden; 3 Umeå Centre for Microbial Research (UCMR), Umeå University, Umeå, Sweden; 4 CRISPR Functional Genomics, SciLifeLab and Department of Medical Biochemistry and Biophysics, Karolinska Institutet, Solna, Sweden; 5 Institute of Pharmacy, Pharmacology and Toxicology, Freie Universität Berlin, Berlin, Germany; 6 Umeå Centre for Electron Microscopy (UCEM), Umeå University, Umeå, Sweden; 7 Biochemical Imaging Centre Umeå (BICU), Umeå University, Umeå, Sweden; University of Pennsylvania, UNITED STATES OF AMERICA

## Abstract

A mechanistic understanding of how intracellular pathogens evade the intrinsic defenses of their host cells could open up intriguing therapeutic opportunities. Here, we applied a genome-wide genetic screening approach to investigate the nature of the defensive host cell death response suppressed by the membrane trafficking modulator CpoS, an effector protein secreted by the obligate intracellular bacterial pathogen *Chlamydia trachomatis*. Initially, this work revealed a CpoS-deficient mutant to exhibit a markedly increased dependence on host cellular synthesis of ceramides, the precursors of complex sphingolipids. Using novel microscopic reporters, we then established CpoS’ role in defense evasion to occur by preserving the integrity of *Chlamydia*’s parasitophorous vacuole (the inclusion) via ensuring an adequate sphingolipid supply. More specifically, we observed CpoS deficiency to destabilize inclusions, initially characterized by a release of individual bacteria into the host cell cytosol, then followed by inclusion rupture concomitant with host cell death. Exogenous addition of sphingosine stabilized CpoS-deficient inclusions, whereas disruption of host cellular ceramide synthesis destabilized wild-type inclusions. In combination, CpoS deficiency and impaired ceramide synthesis – presumably disrupting both *Chlamydia*’s vesicular and non-vesicular sphingolipid supply routes – destabilized inclusions even earlier, resulting in infection clearance and host cell survival rather than host cell death. Overall, this study highlights how the vacuolar pathogen *C. trachomatis* maintains vacuole integrity by ensuring a steady sphingolipid supply, potentially offering inspiration and directions for future therapeutic strategies targeting parasitophorous vacuoles.

## Abstract 2

Intracellular pathogens like Chlamydia trachomatis evade host defenses by preserving vacuole integrity. Using a genome-wide screen, this study shows that the effector CpoS maintains the stability of Chlamydia’s parasitophorous vacuole, and early vacuole destabilization clears infection, highlighting a potential therapeutic target.

## Introduction

To flourish within human cells, intracellular pathogens must adeptly circumvent their host cells’ defenses. Targeting this intricate interplay between host cell-autonomous immunity and pathogen evasion strategies could provide novel therapeutic avenues. Yet, our insufficient understanding of the underlying mechanisms remains a major roadblock. Here, we took a molecular genetic approach to resolve mechanisms of defense evasion in *Chlamydia trachomatis*, a clinically important bacterial pathogen that is exceptionally dependent on its host cells.

Initially identified as the cause of trachoma, a blinding ocular disease that remains a significant public health concern in more than 40 countries [1], *C. trachomatis* is now recognized as a major bacterial agent of sexually transmitted infections, which can inflict enduring harm to reproductive tissues and accounts for about 130 million cases annually [2,3]. *C. trachomatis* exhibits an obligate intracellular lifestyle and a developmental biology characterized by an alternation between two distinct forms, the replicative reticulate body (RB) and the infectious elementary body (EB) [4]. Upon entering a host cell, the EB settles within a membrane-bound vacuole, the inclusion, where it differentiates into the RB form, which then replicates and enlarges the vacuole. At around 24 hrs post-infection (hpi), the RBs begin reverting back into EBs, which are released from the cell at about 48–72 hpi via host cell lysis or inclusion extrusion [5].

Intracellular bacteria employ an arsenal of secreted effector proteins to modulate host cell functions and shape their intracellular niche. In *Chlamydia*, this includes a unique class of effectors, the inclusion membrane proteins (Incs), which the pathogen inserts into the membrane of its vacuole [6]. The recent introduction of tools enabling the molecular genetic manipulation of the pathogen revolutionized our capacity to decipher the critical functions of these effectors [7]. Significantly, these novel approaches revealed the Inc CpoS (also known as CTL0481 or CT229) to evade host cellular immune surveillance [8,9]. Infections with CpoS-deficient mutants lead to a potent activation of the STING/TBK1/IRF3 immune signaling pathway, resulting in increased type I interferon (IFN) production and induction of IFN-stimulated genes [8]. The host cells then also succumb to death prematurely, effectively disrupting bacterial replication and EB formation, and causing an accelerated clearance from the genital tract of experimentally infected mice [8,9]. This defensive host cell death response depends on host cellular protein synthesis, suggesting it is actively triggered by the host cells [8]. CpoS’ interactions with host Rab GTPases were found to be crucial not only in the effector’s ability to modulate membrane trafficking [8,10–12], but also in its role in dampening the induction of the STING/TBK1/IRF3 signaling pathway [11]. However, the defensive cell death response activated in the absence of CpoS was found to only partially rely on STING [8,9], and to not depend on a functional type I IFN response [8], thus cannot be considered a direct result of the enhanced IFN signaling. The nature of the host defense program evaded by CpoS and the reasons for its activation in the absence of the effector therefore remained unclear.

Given the complexities of host-pathogen interactions and built-in redundancies, their mechanistic basis can be difficult to reveal fully when employing genetic approaches targeting the pathogen alone. Hence, we opted to use the powerful but currently underexploited strategy of combining both host- and bacteria-targeting genetic approaches in a genome-wide screening set-up. This allowed unexpected insights into CpoS’ function in the host defense evasion.

## Results

### A CRISPR screen identified host factors contributing to *C. trachomatis* L2-induced cytotoxicity

To identify host genes crucial for the execution of the defensive cell death response suppressed by CpoS, we conducted a CRISPR/Cas9 knockout screen for host gene deficiencies that can protect cells from death triggered by a *C. trachomatis cpoS* null mutant. It is important to note that any deficiencies preventing bacterial entry into cells or significantly perturbing bacterial growth could also be assumed to protect cells from cytotoxicity following exposure to the bacteria. Hence, to aid in pinpointing those genes that contribute to the defensive cell death response specifically, but not to *Chlamydia*-induced cytotoxicity or *Chlamydia* infection generally, we concurrently screened for host gene deficiencies protecting cells from the wild-type strain of *C. trachomatis*.

**Fig 1:**
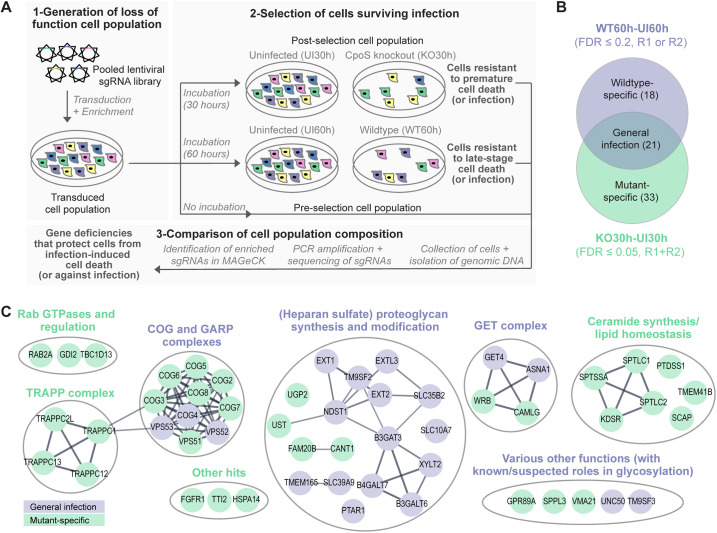
A CRISPR screen identified host factors contributing to *C. trachomatis* L2-induced cytotoxicity. **(A)** CRISPR screening procedure. The screen was conducted in two independent replicates (R1 + R2). **(B)** Venn diagram illustrating the selection of screening hits. **(C)** STRING interaction network displaying interactions among and between “general infection hits” (lilac nodes) and “mutant-specific hits” (green nodes), refined by manual grouping and annotations. Mixed groups were deemed to represent biological processes of general importance for infection (lilac group labels), while groups containing only “mutant-specific hits” were considered to have specific importance during infection with the CpoS-deficient mutant (green group labels). The data underlying this figure can be found in S4 Data.

To this end, we transduced HeLa cells, a human cervical epithelial cell line, with a genome-wide knockout library (Fig 1A). We then infected the cells with wild-type *C. trachomatis* L2/434/Bu (CTL2, WT) or a variant carrying an insertional disruption/knockout of *cpoS* (CTL2-*cpoS*::*cat*, KO). Subsequently, we incubated the cells until a large proportion had died to enrich for cells resistant to killing, that is, cells resistant to either infection or infection-mediated cell death. From cultures infected with CTL2-*cpoS*::*cat,* surviving cells were collected at 30 hpi when about 90% of the cells had died (KO30h) (S1A Fig and S1A Data). Yet, from cultures infected with CTL2*,* surviving cells had to be collected at 60 hpi (WT60h), when only about 75% of the cells had perished, because a later harvesting would have led to significant cell loss during collection due to the fragility of cells containing large inclusions. Uninfected cells were collected alongside the infected cells (UI30h and UI60h). Upon concluding the selection experiments, sgRNA sequencing confirmed a good library representation, with over 99.9% of all library sgRNAs detected in all samples (S1B Fig and S1B Data), and no drastic shifts in population composition (S1C Fig and S1C Data).

We then identified sgRNAs that were positively selected, *i.e.*, sgRNAs that showed significant enrichment in infected compared to uninfected samples and can thus be assumed to target genes promoting infection or infection-mediated cell killing (S2A–S2E Data). By setting a false discovery rate (FDR) of ≤0.05 and an average fold change (FC) of ≥1.25 as cutoffs, we found 54 genes with sgRNAs enriched in KO30h versus UI30h in both replicates of the screen (S1D–S1E Fig and S3A–S3B Data). Using the same criteria, sgRNAs for only three genes showed enrichment in WT60h versus UI60h (S1D–S1E Fig and S3A–S3B Data), aligning with the weaker selection achieved for this strain (S1A Fig and S1A Data). Hence, for the latter comparison, we decided to consider sgRNAs enriched at lower confidence as well, in order to obtain a more comprehensive overview of genes that may contribute more generally to *Chlamydia-*induced cytotoxicity or infection, thereby also facilitating their later exclusion from our analyses focusing on CpoS (as described below).

Consequently, we classified genes targeted by sgRNAs enriched (FC ≥ 1.25) in both KO30h versus UI30h (FDR ≤ 0.05, both replicates) and WT60h versus UI60h (FDR ≤ 0.2, at least one replicate) as “general infection hits”, resulting in 21 genes (Fig 1B and S4A Data). In addition, we identified “mutant-specific hits” as genes whose sgRNAs were enriched in KO30h versus UI30h (FDR ≤ 0.05, both replicates) but not WT60h versus UI60h (FDR ≤ 0.2, any replicate), yielding 33 genes (Fig 1B and S4B Data). While genes with sgRNAs enriched in WT60h versus UI60h but not KO30h versus UI30h could theoretically be considered as “wildtype-specific hits” (Fig 1B and S4C Data), they were not further considered in this study given that they can be expected to be less reliable.

To comprehend the biological roles of the genes targeted by positively selected sgRNAs, we conducted a functional enrichment analysis in g:Profiler [13] using the Reactome pathway database [14]. This revealed that the “general infection hits” were enriched for genes involved in proteoglycan biosynthesis and intra-Golgi trafficking, while the “mutant-specific hits” were enriched for genes involved in various vesicular transport processes and sphingolipid metabolism (S2A–S2B Fig and S4D Data). To gain an even deeper understanding of the major biological processes represented by our hits, we further utilized the STRING database [15] to display a protein interaction network, which we then further refined by manual grouping and annotation based on literature. This in-depth analysis highlighted concrete molecular pathways and complexes in proteoglycan synthesis, vesicular transport, and lipid synthesis to have roles in infection or infection-mediated cell death (Fig 1C and S4E Data). Below, these will be discussed and in part validated and followed up on.

### Deficiencies in heparan sulfate proteoglycan synthesis provide general protection against *C. trachomatis* L2

Since the primary goal of our CRISPR screen was to identify host factors that have specific roles during infection with the CpoS-deficient mutant (CTL2-*cpoS*::*cat*), we aimed to exclude from further in-depth analysis any genes that were either part of the “general infection hits” or involved in the same molecular machineries or pathways. This most notably included genes having key or auxiliary functions in the synthesis of heparan sulfate proteoglycans (HSPGs), cell surface molecules already previously shown to facilitate host cell attachment and invasion by *C. trachomatis* L2 [16–19]. Indeed, this group of genes was especially dominant among our overall pool of hits and included genes encoding all enzymes required for the synthesis of the tetrasaccharide linker in HSPGs (XYLT2, B4GALT7, B3GALT6, B3GAT3 [20]), the enzymes involved in sugar chain elongation (EXTL3, EXT1, EXT2 [20]), proteins involved in glycan chain modifications (NDST1, FAM20B, UST [20,21]), proteins mediating the synthesis, transport, or removal of precursors or side products (SLC35B2, UGP2, CANT1 [20,22,23]), proteins maintaining a functional glycosylation or glycan-modifying machinery (*e.g.*, TM9SF2, TMEM165, SLC10A7, SLC39A9, PTAR1, SPPL3, GRP89A, VMA21 [24–32]), and proteins often found co-enriched in other screens with those acting in HSPG biosynthesis (UNC50 and TM9SF3, *e.g.*, [25,27,33]) (Fig 1C and S3A Fig and S4E Data).

Several other molecular machineries and pathways also included both “mutant-specific hits” and “general infection hits” (Fig 1C and S4E Data), and we thus considered them also more likely to have general roles during *C. trachomatis* L2 infection rather than specific roles related to CpoS. A closer look even suggested that many of these proteins may also have roles in HSPG synthesis. For example, the guided entry of tail-anchored proteins (GET) pathway, which mediates membrane insertion of tail-anchored proteins [34], and the conserved oligomeric Golgi (COG) and Golgi-associated retrograde protein (GARP) complexes, which have roles in membrane trafficking [35,36], may regulate the localization of proteins involved in HSPG synthesis or modification. Indeed, deficiencies in COG and GARP have previously been shown to cause a mislocalization and destabilization of proteins of the glycosylation machinery at the Golgi apparatus [37,38]. To validate this finding, we generated HeLa cells deficient for a key component of the COG complex, COG3 (S3B Fig), and indeed observed them to have HSPG surface levels reduced to a degree similar as seen in cells deficient for EXT1 (S3C Fig and S5A Data). Moreover, infection with wild-type *C. trachomatis* L2 (CTL2) was significantly reduced in COG3-deficient cells, while a much more modest reduction was observed for *C. trachomatis* serovar E (CTE) (S3D Fig and S5B Data), known to invade cells in a less HSPG-dependent manner [39].

In conclusion, this first mining of the data underscored the significance of HSPGs for CTL2 infection, which validated the performance of the screen and at the same time enabled us to focus our further attention on other processes promising specific insights into the function of CpoS.

### Deficiencies in ceramide synthesis provide specific protection against the *cpoS* mutant

**Fig 2:**
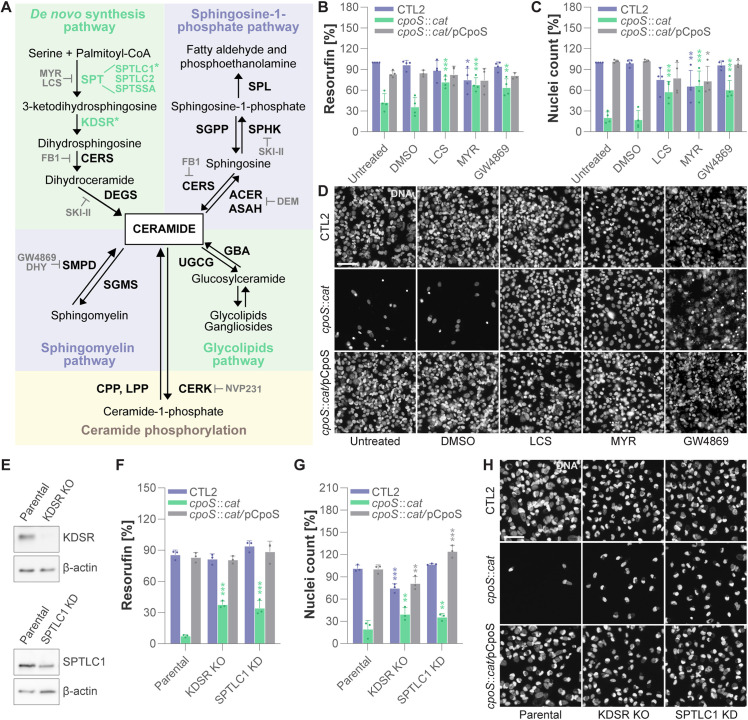
Deficiencies in ceramide synthesis provide specific protection against the *cpoS* mutant. **(A)** Illustration of pathways in ceramide synthesis and metabolism. Enzymes whose deficiency protected cells from *cpoS* mutant-induced toxicity in the screen are highlighted in bold green, pharmacologic inhibitors used in this study in bold gray. Asterisks mark enzymes selected for genetic validation via individual CRISPR/Cas9-mediated gene knockout or knockdown. The names of the inhibitors are given in the main text. Enzymes: ACER/ASAH, ceramidase; CERK, ceramide kinase; CERS, ceramide synthase; CPP, ceramide-1-phosphate phosphatase; DEGS, dihydroceramide desaturase; GBA, glucosylceramidase; KDSR, 3-ketodihydrosphingosine reductase; LPP, lipid phosphate phosphatase; SGMS, sphingomyelin synthase; SGPP, sphingosine-1-phosphate phosphatase; SMPD, sphingomyelinase; SPHK, sphingosine kinase; SPL, sphingosine phosphate lyase; SPT, serine palmitoyl transferase; UGCG, UDP-glucose ceramide glycosyltransferase. **(B–D)** Selected inhibitors targeting sphingolipid metabolism protected cells against *cpoS* mutant-induced death. HeLa cells were treated with the indicated inhibitors (LCS, 125 µM; MYR, 1 µM; GW4869, 12.5 µM) or solvent only (DMSO), and were parallelly infected with the indicated strains (5 IFU/cell). Resorufin fluorescence (B) and nuclei count (C) at 25.5 hpi are displayed normalized to a CTL2-infected untreated control (mean ± SD, *n* = 4, two-way ANOVA with Dunnett’s post-hoc test; for each strain, indicated are significant differences compared to the DMSO control). (D) Representative images of DNA (Hoechst) staining (scale = 80 µm). **(E)** western blot analysis confirming the depletion (knockdown, KD) of SPTLC1 and absence (knockout, KO) of KDSR in the respective HeLa cell lines. **(F–H)** Depletion of SPTLC1 or deficiency in KDSR partially protected cells against *cpoS* mutant-induced death. The indicated HeLa cell lines were infected with the indicated strains (5 IFU/cell). Resorufin fluorescence (F) and nuclei count (G) at 24 hpi are displayed normalized to an uninfected control (mean ± SD, *n* = 3, one-way ANOVA with Dunnett’s post-hoc test; for each strain, indicated are significant differences compared to the parental (wild-type) cells). (H) Representative images of DNA (Hoechst) staining (scale = 80 µm). The data underlying this figure can be found in S6 Data.

While we observed several host gene deficiencies to provide specific protection against CTL2-*cpoS*::*cat* in the CRISPR screen, they unexpectedly did not affect genes with known roles in immune signaling or regulated cell death. Instead, we found such impacting the Transport Protein Particle (TRAPP), a multi-subunit tethering complex involved in the vesicular trafficking between the endoplasmic reticulum (ER) and the Golgi apparatus [40], as well as the Rab GTPase RAB2A, also active in ER-Golgi trafficking [41], and GDI2 and TBC1D13, regulators of Rab protein function [41,42] (Fig 1C and S4E Data). Intriguingly, we also found specific protection to be conferred by deficiencies in the *de novo* synthesis pathway of ceramides, in particular such affecting the first enzymatic step, catalyzed by serine palmitoyltransferase (SPT; composed of SPTLC1, SPTLC2, and SPTSSA), or the second enzymatic step, catalyzed by 3-ketodihydrosphingosine reductase (KDSR) (Figs 1C and 2A and S4E Data). While all these screening hits deserve further investigations, in the present study, we decided to follow-up on the observations pertaining to ceramide synthesis, because they connected to our previous finding that CpoS, via its interactions with host Rab GTPases, mediates the vesicular transport of ceramide-derived sphingolipids to *C. trachomatis* L2 inclusions [11].

To validate the screening hits in ceramide synthesis, we initially employed a pharmacologic approach leveraging the availability of well-established SPT inhibitors [L-cycloserine (LCS) and myriocin (MYR)] (Fig 2A). We additionally tested the effect of inhibitors targeting other enzymes in ceramide metabolism, including ceramide synthase [fumonisin B1 (FB1)], neutral sphingomyelinase [GW4869], acid sphingomyelinase [desipramine hydrochloride (DHY)], alkaline ceramidase [D-erythro-MAPP (DEM)], and ceramide kinase [NVP231] (Fig 2A). We treated HeLa cells with the respective inhibitors at non-toxic concentrations (S4A Fig and S6A Data) and simultaneously infected them with either CTL2 or CTL2-*cpoS*::*cat*. At 24 hpi, we quantified host cell viability based on the ability of viable cells to convert resazurin into fluorescent resorufin and based on the number of remaining cell nuclei. In agreement with the findings from the CRISPR screen, the SPT inhibitors LCS and MYR partially protected cells from death after infection with the *cpoS* mutant (S4B–S4C Fig and S6B–S6C Data). Additionally, our analyses indicated reduced cell death also in cultures treated with GW4869 (S4B–S4C Fig and S6B–S6C Data), although it should be noted that we observed some precipitation of GW4869, which may potentially have affected fluorescence recordings. The experiments with these three inhibitors (LCS, MYR, and GW4869) were subsequently repeated including as additional control also infections with a complemented mutant strain, *i.e.*, a derivative of CTL2-*cpoS*::*cat* transformed with a plasmid driving expression of CpoS (CTL2-*cpoS*::*cat*/pCpoS). These additional analyses confirmed the inhibitor effects on *cpoS* mutant-induced cell death and demonstrated that complementation restored wild-type phenotypes (Fig 2B–2D and S6D–S6E Data).

To obtain genetic confirmation of our findings, we subsequently used CRISPR/Cas9 to generate a cell line depleted (knockdown, KD) for SPTLC1 and a cell line deficient (knockout, KO) for KDSR (Fig 2E). In agreement with the findings from the CRISPR screen and the experiments with the SPT inhibitors, these cell lines were partially protected against *cpoS* mutant-induced cell death (Fig 2F–2H and S6F–S6G Data).

In summary, these findings indicated that deficiencies in or the inhibition of ceramide-producing enzymatic reactions can protect cells from death following exposure to the *cpoS* mutant.

### CpoS deficiency causes potentially cytotoxic alterations in sphingolipid metabolite levels

Ceramides and derived metabolites are not only precursors for complex sphingolipids, such as sphingomyelin, but function also as signaling molecules with key roles in cellular life/death decisions [43]. Therefore, it seemed plausible that a deficiency in *cpoS* may instigate host cell death by disturbing metabolite balance, for instance, by resulting in a cytotoxic accumulation of ceramides, an effect that impairments in the ceramide synthesis pathway may potentially mitigate. To investigate this possibility, we employed mass spectrometry to quantify the intracellular levels of 31 sphingolipid metabolites, encompassing a variety of common sphingoid bases, dihydroceramides, ceramides, dihydrosphingomyelins, sphingomyelins, hexosylceramides, and lactosylceramides. We focused on an early time point during infection, more specifically 14 hpi, which is just before premature host cell death becomes apparent in cultures infected with CTL2-*cpoS*::*cat*.

**Fig 3:**
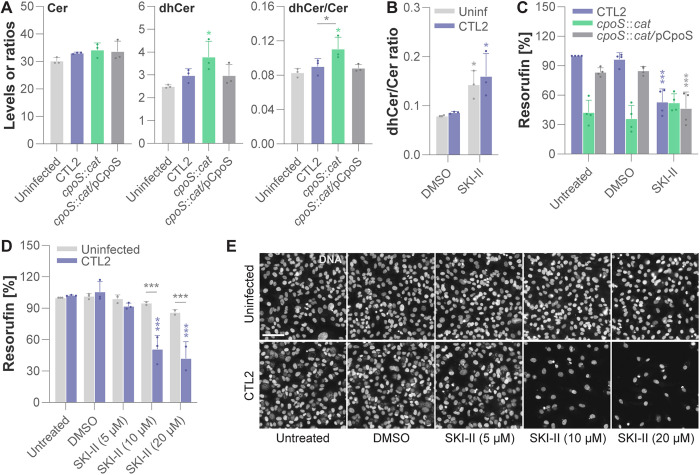
CpoS deficiency causes potentially cytotoxic alterations in sphingolipid metabolite levels. **(A)** Infection with the *cpoS* mutant enhanced the dihydroceramide (dhCer) to ceramide (Cer) ratio in infected cells. Cell extracts from HeLa cells infected with the indicated strains (10 IFU/cell) were prepared at 14 hpi, and the indicated lipids were quantified by LC–MS/MS. Shown are selected metabolite levels (expressed as “fmol/pmol total sphingolipids”) or ratios (mean ± SD, *n* = 3, one-way ANOVA with Tukey’s post-hoc test; if not specified otherwise, indicated are significant differences compared to uninfected cells). Full data are presented in S5 Fig. **(B)** Mass spectrometric analysis confirming SKI-II to increase the dhCer to Cer ratio. Cell extracts from uninfected and CTL2-infected (5 IFU/cell) HeLa cells, treated with SKI-II (6.25 µM) at 0 hpi, were prepared at 14 hpi, and the indicated lipids were quantified by LC–MS/MS and displayed as ratio (mean ± SD, *n* = 3, two-way ANOVA with Sidak’s post-hoc test; indicated are significant differences compared to the DMSO control). **(C)** SKI-II induced death in cells infected with CTL2. HeLa cells were treated with SKI-II (6.25 µM) or solvent only (DMSO) and were parallelly infected with the indicated strains (5 IFU/cell). Resorufin fluorescence at 25.5 hpi is displayed normalized to a CTL2-infected untreated control (mean ± SD, *n* = 4, two-way ANOVA with Sidak’s post-hoc test; for each strain, indicated are significant differences compared to the DMSO control). **(D–E)** SKI-II induced death specifically in infected but not uninfected cells. HeLa cells were treated with the indicated concentrations of SKI-II or with solvent only (DMSO) and were parallelly infected with CTL2 (5 IFU/cell) or left uninfected. (D) Resorufin fluorescence at 34 hpi is displayed normalized to the uninfected untreated control (mean ± SD, *n* = 3, two-way ANOVA with Sidak’s post-hoc test; if not specified otherwise, indicated are for each infection condition significant differences compared to the DMSO control). (E) Representative images of DNA (Hoechst) staining (scale = 80 µm). The data underlying this figure can be found in S7 Data.

Interestingly, at this early time after infection, we did not detect any remarkable differences in the metabolite levels between uninfected cells and cells infected with either CTL2 or CTL2-*cpoS*::*cat*/pCpoS (Fig 3A and S5 Fig and S7A Data). Moreover, most metabolite levels remained similar also during infection with CTL2-*cpoS*::*cat*. Notably, ceramide levels were not significantly increased during infection with the CpoS-deficient mutant. However, we observed an increase in the levels of dihydroceramides and thus also in the dihydroceramide to ceramide ratio (Fig 3A and S5 Fig and S7A Data), an alteration previously reported to be cytotoxic [44].

To understand if the observed metabolic changes could contribute to the premature cell death seen during infection with CTL2-*cpoS*::*cat*, we employed the pharmacologic inhibitor SKI-II. This molecule inhibits sphingosine kinase (SPHK), but was recently shown to also inhibit dihydroceramide desaturase (DEGS), thereby increasing the dihydroceramide to ceramide ratio in treated cells (Fig 2A) [45]. Indeed, mass spectrometric analyses confirmed that SKI-II causes an accumulation of dihydroceramides in both uninfected cells and cells infected with CTL2 (Fig 3B and S7B Data). Intriguingly, the inhibitor also induced cell death in cultures infected with CTL2 or CTL2-*cpoS*::*cat*/pCpoS while not further augmenting death in cultures infected with CTL2-*cpoS*::*cat* (Fig 3C and S7C Data). This toxicity of SKI-II towards CTL2-infected cells was seen even at concentrations that were non-toxic to uninfected cells (Fig 3D–3E and S7D Data).

In summary, our findings indicated an increased dihydroceramide to ceramide ratio in cells infected with the *cpoS* mutant, a metabolic alteration that could potentially contribute to the observed premature host cell death.

### The *cpoS* mutant displays an increased dependence on host *de novo* ceramide synthesis

Given the observed effects of the inhibitors on the viability of infected cells, we hypothesized that SKI-II would effectively eradicate CTL2 infection from infected cultures, while the inhibitors MYR, LCS, and GW4869, which can be expected to block formation of dihydroceramides and/or ceramides and partially blocked *cpoS* mutant-induced premature cell death (Fig 2B–2D and S4B–S4C Fig and S6B–S6E Data), may restore the intracellular growth of CTL2-*cpoS*::*cat*.

**Fig 4:**
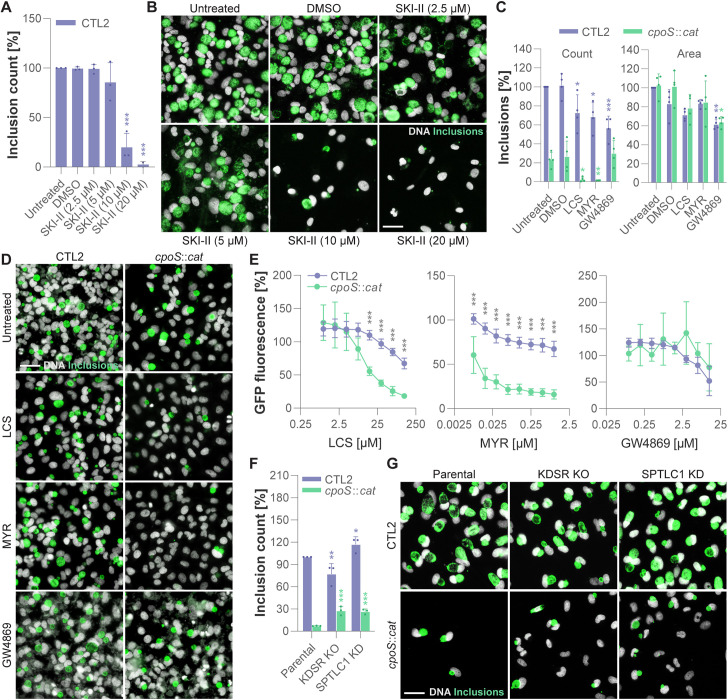
The *cpoS* mutant displays an increased dependence on host *de novo* ceramide synthesis. **(A–B)** SKI-II treatment eradicated infected cells from cultures infected with CTL2. HeLa cells were treated with the indicated concentrations of SKI-II or with solvent only (DMSO), and parallelly infected with a GFP-expressing derivative of CTL2 (5 IFU/cell). Bacterial inclusions were detected microscopically at 34 hpi. (A) Inclusion numbers displayed relative to CTL2 inclusion count in the untreated control (mean ± SD, *n* = 2–3, one-way ANOVA with Dunnett’s post-hoc test; indicated are significant differences compared to the DMSO control). (B) Representative images showing inclusions (GFP) and DNA (Hoechst) staining (scale = 40 µm). **(C–D)** MYR and LCS, but not GW4869, reduced inclusion count after infection with the *cpoS* mutant. HeLa cells were treated with the indicated inhibitors (LCS, 125 µM; MYR, 1 µM; GW4869, 12.5 µM) or solvent only (DMSO), and parallelly infected with GFP-expressing derivatives of the indicated strains (1 IFU/cell). Bacterial inclusions were detected microscopically at 26.5 hpi. (C) Data displayed as percentage relative to inclusion count and average inclusion area detected for CTL2 in the absence of inhibitors (mean ± SD, *n* = 4, one-way ANOVA with Dunnett’s post-hoc test; for each strain, indicated are significant differences compared to the DMSO control). (D) Representative images showing inclusions (GFP) and DNA (Hoechst) staining (scale = 40 µm). **(E)** Differential susceptibility of *cpoS* mutant and wild-type bacteria to SPT inhibition. HeLa cells were treated with the indicated inhibitors and parallelly infected with GFP-expressing derivatives of the indicated strains (1 IFU/cell). GFP fluorescence measured at 25.5 hpi is displayed relative to fluorescence detected for the respective strain in the absence of inhibitors (mean ± SD, *n* = 3–4, 2-way ANOVA with Sidak’s post-hoc test; for each concentration, indicated are significant differences between the two strains). **(F–G)** Depletion of SPTLC1 or deficiency in KDSR partially restored inclusion count after infection with the *cpoS* mutant but increased the occurrence of inclusion-free cells. The indicated HeLa cells lines were infected with the indicated strains (5 IFU/cell). (F) Inclusion numbers detected at 24 hpi displayed normalized to the number of inclusions detected for CTL2 in the parental cells (mean ± SD, *n* = 3, two-way ANOVA with Dunnett’s post-hoc test; for each strain, indicated are significant differences compared to the parental cells). (G) Representative images showing inclusions (detected by immunofluorescence staining of the bacterial protein Slc1) and DNA (Hoechst) staining (scale = 40 µm). The data underlying this figure can be found in S8 Data.

Hence, we treated HeLa cells with the inhibitors and concurrently infected them with GFP-expressing derivatives of CTL2 or CTL2-*cpoS*::*cat*, followed by microscopic detection of bacterial inclusions. In cultures infected with CTL2, SKI-II indeed caused a large drop in the numbers of both cells and inclusions, and the majority of cells surviving treatment did not harbor bacteria, in line with the inhibitor selectively killing infected but not uninfected cells (Fig 4A–4B and S8A Data). On the contrary, MYR, LCS, and GW4869 caused only minor reductions in the counts of both cells and CTL2 inclusions, as well as the size of the inclusions (Fig 4C–4D and S8B Data). In cultures infected with CTL2-*cpoS*::*cat*, the total number of inclusions detected in the absence of inhibitors was strongly reduced when compared to CTL2 infection, which was expected given the cell loss due to premature host cell death (Fig 4C–4D and S8B Data). Unexpectedly, LCS and MYR, while protecting cells infected with CTL2-*cpoS*::*cat* from death, not only failed to restore the growth of the bacteria but almost entirely eradicated the infection from treated cultures, with the majority of the remaining cells harboring no inclusions (Fig 4C–4D and S8B Data). In contrast, the presence of GW4869 did not modify the number of CTL2-*cpoS*::*cat* inclusions (Fig 4C–4D and S8B Data). A comparison of growth inhibition across a range of inhibitor concentrations, using GFP fluorescence derived from GFP-expressing strains as a measure for bacterial growth, confirmed that the *cpoS* mutant was considerably more dependent on SPT function (Fig 4E and S8C Data). Corresponding experiments with GW4869 suggested both strains respond similarly to this inhibitor (Fig 4E and S8C Data).

Interestingly, in the cell lines depleted for SPTLC1 or deficient for KDSR, a slightly increased number of inclusions was detected after infection with CTL2-*cpoS*::*cat* (Fig 4F–4G and S8D Data)*.* However, it was also apparent that a high proportion of the remaining cells in those cultures, *i.e.,* the cells that were protected from the premature host cell death, were inclusion-free, in line with SPTLC1 depletion and KDSR deficiency having a growth-suppressing effect on CTL2-*cpoS*::*cat* (Fig 4G). It is likely that this growth suppression resulting from impairments in SPT or KDSR function contributes to the observed cytoprotective effects.

Previous research has shown that *C. trachomatis* L2 has a requirement for host-derived sphingomyelin [46] and can acquire the lipid in two ways: First, by redirecting sphingolipid-laden membrane vesicles to the inclusion [47]; second, by recruiting the host ceramide transfer protein CERT to inclusion-ER contact sites, facilitating non-vesicular acquisition of ceramides then converted to sphingomyelin at the inclusion [48]. We previously reported CERT recruitment, which others have shown to be mediated by the inclusion membrane protein IncD [49,50], to be unaffected by CpoS deficiency [11]. On the contrary, the vesicular transport of sphingomyelin to inclusions was compromised in the absence of CpoS [11]. Hence, we hypothesized that the *cpoS* mutant’s heightened dependence on *de novo* ceramide synthesis could be attributed to its reduced capacity to utilize the already available sphingolipid pool in the host cell, possibly resulting in a higher dependence on the CERT-mediated transport route.

Indeed, in the presence of golgicide A, an inhibitor known to block vesicular transport of Golgi-derived sphingomyelin to the inclusion [48], the dependence of CTL2 on host *de novo* ceramide synthesis intensified (S6A Fig and S8E Data). Moreover, the CRISPR screening data suggested a trend of CERT deficiency protecting cells from *cpoS* mutant-induced cell death, while interestingly enhancing the susceptibility of cells to CTL2-induced cytotoxicity (S6B Fig and S8F Data). Similar observations were made with a HeLa cell line depleted for CERT (S6C-S6D Fig and S8G Data). Moreover, despite being cytoprotective, CERT depletion did not significantly increase the number of inclusions detectable after infection with CTL2-*cpoS*::*cat* and many cells in cultures infected with this strain were inclusion-free, suggesting that CERT depletion, similar to SPTLC1 depletion and KDSR deficiency, may have a growth-suppressing rather than a growth-restoring effect on CTL2-*cpoS*::*cat* (S6E–S6F Fig and S8H Data).

In summary, our research indicated that the cytoprotective effects resulting from impairments in SPT or KDSR function in cultures infected with CTL2-*cpoS*::*cat* may not be explained by an effect on dihydroceramide levels alone, as CpoS deficiency heightens the bacteria’s reliance on host cellular ceramide synthesis for inclusion formation and/or growth.

### Novel reporters for inclusion damage revealed instability of CpoS-deficient inclusions

We next sought to obtain a deeper mechanistic understanding of the ways by which sphingolipid deprivation can curb the growth of CTL2-*cpoS*::*cat*. It is important noting that CpoS deficiency has previously been proposed to cause a premature rupture of the inclusion [9]. Moreover, SPT inhibition has been suggested to destabilize CTL2 inclusions [51]. Hence, assuming that a potential instability of CpoS-deficient inclusions could stem from the mutant’s reduced ability to acquire sphingolipids via vesicular transport [11], we may further assume that SPT inhibition could aggravate and accelerate the destabilization of CpoS-deficient inclusions. This may then possibly modify the outcome for the host cell from death to infection clearance. However, live cell imaging suggested that CpoS-deficient inclusions rupture only at the onset of cell death [52], leaving unclear if CpoS deficiency indeed causes damage that later cumulates into rupture, possibly triggering cell death, or if described ruptures were merely a consequence of cell death.

**Fig 5:**
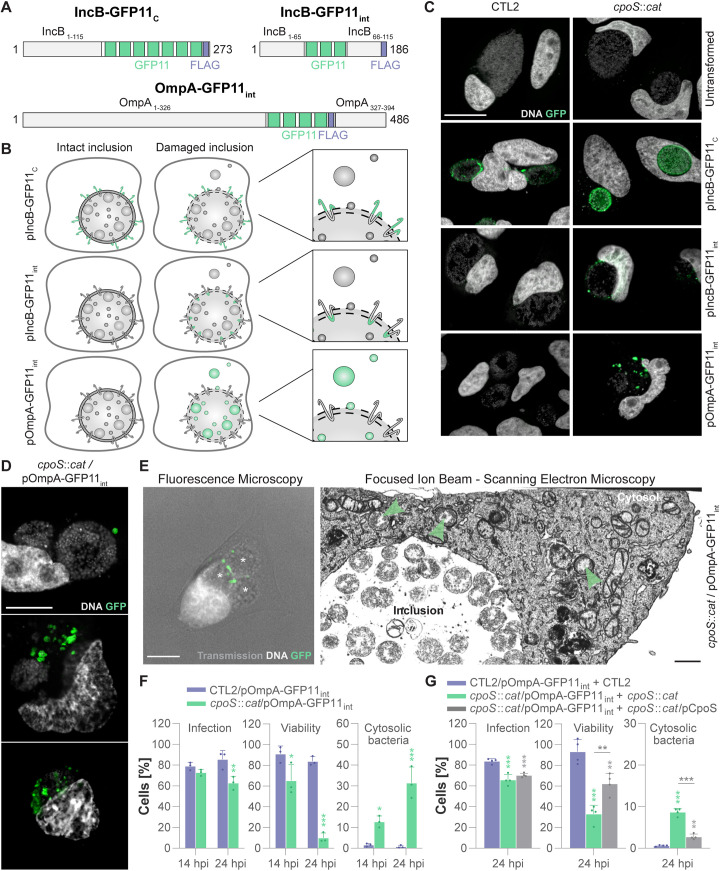
Novel reporters for inclusion damage revealed instability of CpoS-deficient inclusions. **(A)** Composition of the three GFP11-tagged constructs. **(B)** The principle of detecting inclusion damage using the split-GFP approach. **(C)** Fluorescence microscopic detection of inclusion damage during infection with CTL2-*cpoS*::*cat*. HeLa cells were transfected with a plasmid driving GFP1–10 expression, infected with the indicated strains (5 IFU/cell), and then fixed, stained (DNA (Hoechst) staining), and imaged at 26 hpi (scale = 20 µm). **(D)** Confocal fluorescence microscopic images displaying different grades of inclusion damage. GFP1–10-expressing HeLa cells were infected with the indicated strain (10 IFU/cell) and then fixed, stained (DNA (Hoechst) staining), and imaged at 24 hpi (scale = 10 µm). **(E)** FIB-SEM analysis validating inclusion damage at the ultrastructural level. GFP1–10-expressing HeLa cells were infected with the indicated strain and fixed at 24 hpi. A cell containing green-fluorescent bacteria was identified by fluorescence microscopy (left, scale = 10 µm, asterisks highlight inclusions) and subjected to FIB-SEM analysis (right, scale = 1 µm, one selected slice of the volume shown in S1 Movie, green arrows highlight bacteria in the host cell cytosol). **(F)** Quantitative analysis of inclusion damage in cells infected with the *cpoS* mutant. GFP10-expressing HeLa cells were infected with the indicated strains (10 IFU/cell) and then fixed, stained, and imaged at the indicated time points. The percentage of infected cells, surviving cells, and infected cells containing cytosolic bacteria was determined by manual image analysis (mean ± SD, *n* = 3, at least 100 cells per condition and replicate counted, 2-way ANOVA with Sidak’s post-hoc test; for each time point, indicated are significant differences compared to CTL2/pOmpA-GFP11_int_). **(G)** Complementation of the *cpoS* mutant restored inclusion integrity. GFP10-expressing HeLa cells were co-infected with the indicated strains (5 IFU/cell of each strain) and then fixed, stained, and imaged at 24 hpi. The percentage of infected cells, surviving cells, and infected cells containing cytosolic bacteria was determined by manual image analysis (mean ± SD, *n* = 4, at least 200 cells per condition and replicate counted, one-way ANOVA with Tukey’s post-hoc test; if not indicated otherwise, indicated are significant differences compared to CTL2/pOmpA-GFP11_int_ + CTL2). Note that due to the fusogenic nature of inclusions, co-infection results in mixed inclusions containing both of the co-infecting strains. The data underlying this figure can be found in S9 Data.

Clarifying this question requires tools enabling the detection of early forms of inclusion membrane damage. Therefore, we developed fluorescence microscopic reporters that can visualize compromised inclusion membranes and bacteria released from damaged inclusions. These reporters employ the split-GFP principle, where the N-terminal part of GFP (GFP1–10) and the C-terminal part (GFP11) are expressed separately but can associate to form a functional fluorescent protein upon co-localization [53]. Previously, the system was successfully applied to detect secretion of *C. trachomatis* effectors or their insertion into the inclusion membrane [54]. Here, we used a modified approach. We generated derivatives of CTL2 and CTL2-*cpoS*::*cat* expressing distinct variants of the Inc protein IncB or the *Chlamydia* outer membrane protein OmpA tagged with both FLAG and GFP11 (Fig 5A). In IncB-GFP11_c_, multiple copies of GFP11 were placed at IncB’s C-terminus, which is exposed to the host cell cytosol during infection. In IncB-GFP11_int_, the GFP11 copies were placed between IncB’s two transmembrane domains, a region presumed to face the inclusion lumen, while in OmpA-GFP11_int_, they were inserted into the predicted surface-exposed loop region between OmpA’s β-strands 11 and 12. Hence, during infection, the GFP11-tags of the constructs are expected to be displayed at the inclusion membrane, facing either towards the host cell cytosol (IncB-GFP11_c_) or the inclusion lumen (IncB-GFP11_int_), or at the surface of the bacteria (OmpA-GFP11_int_). These constructs can thus serve as positive control for the split-GFP system (IncB-GFP11_c_) or facilitate the detection of damaged inclusion membranes (IncB-GFP11_int_) or bacteria exposed to the host cell cytosol (OmpA-GFP11_int_) (Fig 5B).

In infected HeLa cells, all three constructs were expressed and localized correctly to either the inclusion membrane or the bacteria (S7A Fig). In cells expressing cytosolic GFP1–10, IncB-GFP11_c_ at the membrane of CTL2 inclusions became green fluorescent (S7B Fig). In contrast, no green fluorescence was observed in cells infected with CTL2 expressing IncB-GFP11_int_ or OmpA-GFP11_int_ (S7B Fig), consistent with the barrier function of the inclusion membrane. Critically, upon treating cells with membrane-permeabilizing agents, such as digitonin or high concentrations of DMSO, green-fluorescent spots emerged at IncB-GFP11_int_-positive inclusion membranes (S7C Fig). Moreover, inclusions of OmpA-GFP11_int_-expressing bacteria displayed green fluorescence post-treatment (S7C Fig). This fluorescence was faint and diffuse, implying a rupture of both the inclusion and bacterial membranes. With shorter treatment durations, individual green-fluorescent bacteria could still be discerned within some of the inclusions (S7D Fig).

Intriguingly, green-fluorescent spots, indicative of membrane damage, were also observed at CpoS-deficient IncB-GFP11_int_-positive membranes in the absence of permeabilizing agents (Fig 5C). Moreover, a subset of cells infected with OmpA-GFP11_int-_expressing CTL2-*cpoS*::*cat* harbored green-fluorescent bacteria (Fig 5C), often adjacent to seemingly intact non-fluorescent inclusions, likely representing early signs of inclusion damage (Fig 5D). Inclusion rupture, resulting in the majority of the bacteria exhibiting green fluorescence, was observed occasionally, but typically only in cells that appeared to be in the process of dying (Fig 5D). The release of CTL2-*cpoS*::*cat* into the host cell cytosol could further be confirmed at the ultrastructural level, when we selected cells with green-fluorescent bacteria for focused ion beam scanning electron microscopy (FIB-SEM), a technique that can record volumes of infected cells (Fig 5E). Moreover, a quantitative analysis by fluorescence microscopy revealed that at 14 hpi, coinciding with the onset of cell death in cultures infected with CTL2-*cpoS*::*cat*, and at 24 hpi, when extensive cell death was observed, about 12.7% and 31.4% of the infected cells, respectively, contained cytosolic bacteria (Fig 5F and S9A Data). Critically, this analysis also confirmed the near absence of cytosolic bacteria at these times during infection with CTL2. Furthermore, complementation of the *cpoS* mutant restored inclusion stability (Fig 5G and S9B Data).

In summary, our novel microscopic reporters, which enable the detection of early signs of inclusion damage and cytosolic *Chlamydia*, provided evidence that inclusions formed by CTL2 are usually stable and that CpoS indeed plays a role in preserving inclusion integrity, as its absence leads to subtle forms of damage preceding inclusion rupture and cell death.

### Early destabilization of inclusions clears infection while keeping host cells alive

**Fig 6:**
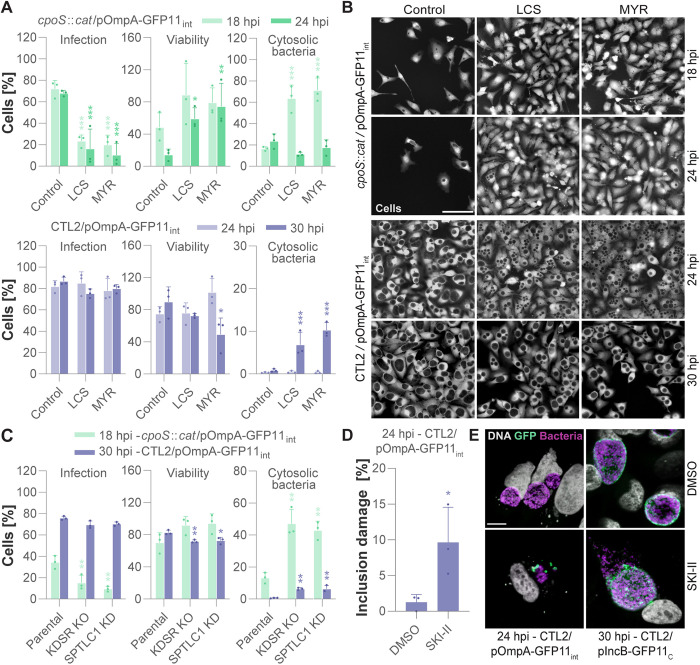
Early destabilization of inclusions clears infection while keeping host cells alive. **(A–B)** Destabilizing effect of SPT inhibition on inclusions. GFP10-expressing HeLa cells were infected with the indicated strains (5 IFU/cell) and parallelly treated with MYR (10 µM) or LCS (125 µM) or left untreated (control). Cells were fixed, stained, and imaged at the indicated time points. (A) The percentage of infected cells, surviving cells, and infected cells containing cytosolic bacteria was determined by manual image analysis (mean ± SD, *n* = 3, at least 100 cells per condition and replicate counted, 2-way ANOVA with Sidak’s post-hoc test; for each time point, indicated are significant differences compared to the untreated control). (B) Representative images of whole cell (HCS) staining (scale = 100 µm). **(C)** Inclusion-destabilizing effects of SPTLC1 depletion or KDSR deficiency. The indicated cell lines were transfected with a GFP1–10-expressing plasmid and then infected with the indicated strains. Cells were fixed, stained, and imaged at the indicated time points. The percentage of infected cells, surviving cells, and infected cells containing cytosolic bacteria was determined by manual image analysis (mean ± SD, *n* = 3, at least 200 cells per condition and replicate counted, one-way ANOVA with Dunnett’s post-hoc test; for each time point (and strain), indicated are significant differences compared to the parental cells). **(D–E)** Treatment with SKI-II destabilizes CTL2 inclusions. GFP10-expressing HeLa cells were infected with the indicated strains (5 IFU/cell) and parallelly treated with SKI-II (10 µM) or solvent-only (DMSO). Cells were fixed at 24 hpi or 30 hpi, stained (Hoechst, DNA; Slc1, bacteria), and imaged. (D) The percentage of infected cells displaying inclusion damage (ruptured inclusions or early damage indicated by individual cytosolic bacteria) was determined by manual image analysis (mean ± SD, *n* = 3, at least 200 cells per condition and replicate counted, unpaired *t* test). (E) Representative images displaying cytosolic bacteria and inclusion rupture (scale = 10 µm). The data underlying this figure can be found in S10 Data.

After having confirmed that CpoS-deficient inclusions are unstable, we sought to investigate whether SPT inhibition indeed exacerbates inclusion damage. We infected GFP1–10-expressing HeLa cells with OmpA-GFP11_int_-expressing CTL2-*cpoS*::*cat* and parallelly treated them with MYR or LCS. In line with our previous findings, we observed robust protection from cell death and a substantial decrease in the proportion of cells containing inclusions at 24 hpi (Fig 6A–6B and S10A Data). Unexpectedly, when observed at this time, the percentage of infected cells containing cytosolic bacteria appeared unaffected by the presence of SPT inhibitors (Fig 6A and S10A Data). However, when we quantified inclusion damage at an earlier time point, at 18 hpi, we observed a strong inclusion-destabilizing effect due to SPT inhibition, with up to 70% of the treated infected cells harboring cytosolic bacteria (Fig 6A and S10A Data). Collectively, these findings confirmed that SPT inhibition aggravates the instability of CpoS-deficient inclusions, with a majority of the inclusions becoming destabilized early on, apparently leading to infection clearance rather than host cell death.

When GFP1–10-expressing HeLa cells were infected with CpoS-proficient OmpA-GFP11_int_-expressing CTL2 and parallelly treated with MYR or LCS, we observed inclusion destabilization only at later stages of infection. Indeed, at 24 hpi, cytosolic bacteria were undetectable (Fig 6A and S10A Data), although we observed an also previously reported [51] inclusion fusion defect in inhibitor-treated cells, as indicated by a high proportion of cells containing multiple inclusions (Fig 6B). Yet, at 30 hpi, cytosolic bacteria were detectable in a small fraction of cells treated with SPT inhibitors, which in cultures treated with MYR also coincided with a slight increase in host cell death (Fig 6A and S10A Data). Overall, this implies that CpoS-proficient inclusions exhibit greater resistance to the destabilizing effects of SPT inhibition. Moreover, destabilization at later stages seems to be linked to cell death, partially mirroring the phenotype of CpoS deficiency.

In the cell lines depleted for SPTLC1 or deficient for KDSR we made similar observations, including aggravated destabilization of CTL2-*cpoS*::*cat* inclusions at 18 hpi, as well as some inclusion destabilization and host cell death induction at 30 hpi with CTL2 (Fig 6C and S10B Data). Moreover, as DEGS inhibition by SKI-II can also be expected to curb sphingolipid supply, we were not surprised to see that this inhibitor also destabilized CTL2 inclusions (Fig 6D–6E and S10C Data), likely explaining its selective toxicity towards infected cells.

Taken together, these findings validate that impairments in the ceramide synthesis pathway cause inclusion destabilization and demonstrate that they in combination with CpoS deficiency, presumably disrupting both sphingolipid supply routes simultaneously, destabilize inclusions at an early infection stage, thereby clearing infection while maintaining the host cells alive.

### Supplementation with sphingosine stabilizes CpoS-deficient inclusions

**Fig 7:**
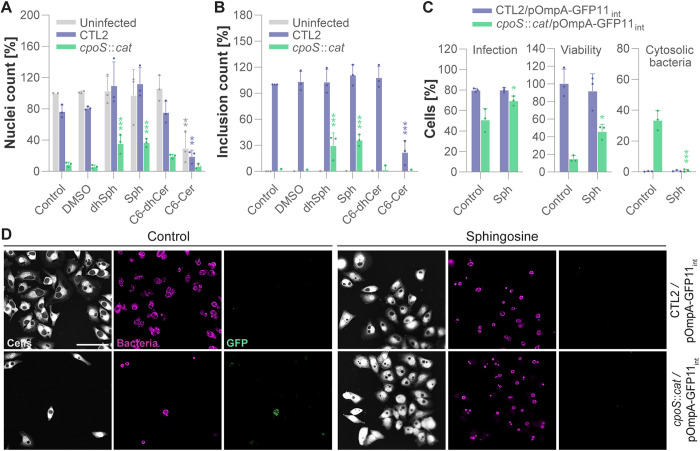
Supplementation with sphingosine stabilizes CpoS-deficient inclusions. **(A–B)** Supplementation of growth media with sphingoid bases protected cells from *cpoS* mutant-induced death and restored bacterial growth. HeLa cells were infected with the indicated strains (4 IFU/cell), and parallelly treated with 5 µM of the indicated metabolites (dhSph, dihydrosphingosine; Sph, sphingosine; C6-dhCer, C6-dihydroceramide; C6-Cer, C6-ceramide; DMSO, solvent only) or left untreated (control). Nuclei count (A) at 25.5 hpi is displayed normalized to the uninfected untreated control, while inclusion count (B) is displayed normalized to the CTL2-infected untreated control (mean ± SD, *n* = 3, one-way ANOVA with Dunnett’s post-hoc test; for each infection condition, indicated are significant differences compared to the DMSO control). **(C–D)** Stabilizing effect of sphingosine on CpoS-deficient inclusions. GFP10-expressing HeLa cells were infected with the indicated strains (5 IFU/cell) and parallelly treated with 5 µM sphingosine (Sph) or left untreated (control). Cells were fixed, stained (HCS, cells; Slc1, bacteria), and imaged at 24 hpi. (C) The percentage of infected cells, surviving cells, and infected cells containing cytosolic bacteria was determined by manual image analysis (mean ± SD, *n* = 3, at least 100 cells per condition and replicate counted, 2-way ANOVA with Sidak’s post-hoc test; for each strain, indicated are significant differences compared to the untreated control). (D) Representative images (scale = 100 µm). The data underlying this figure can be found in S11 Data.

To confirm that the observed destabilization of CpoS-deficient inclusions resulted from diminished sphingolipid acquisition, we provided cells with an excess of sphingolipid metabolites. The addition of C6-D-erythro-ceramide (C6-Cer) to the growth medium proved highly toxic to both infected and uninfected cells (Fig 7A and S11A Data), aligning with the pro-death signaling function of ceramides [43]. Interestingly, C6-D-erytho-dihydroceramide (C6-dhCer) showed no significant toxicity in this assay, which, however, may be explained by the observation that such synthetic cell-permeable short-chain dihydroceramides are generally less toxic than their natural long-chain counterparts [55]. Notably, the addition of D-erythro-sphingosine (Sph) or D-erythro-dihydrosphingosine (dhSph) protected cells from *cpoS* mutant-induced cell death and restored the growth of the mutant to a significant extent (Fig 7A–7B and S11A–S11B Data). The addition of sphingosine also stabilized CpoS-deficient inclusions (Fig 7C–7D and S11C Data).

Collectively, the findings obtained in this study thus underscore CpoS’ role in preserving the intracellular hideout of *C. trachomatis* L2 by ensuring an adequate sphingolipid supply.

## Discussion

A mechanistic understanding of the virulence strategies that allow intracellular pathogens to bypass the defenses of their host cells could open up intriguing therapeutic opportunities. CpoS deficiency in *C. trachomatis* has previously been shown to result in an enhanced induction of immune signaling and premature host cell death, effectively disrupting the intracellular replication of the pathogen [8,9]. Therefore, our initial motivation for implementing a CRISPR screen was to identify the host factors that participate in the immune recognition of CpoS-deficient bacteria and/or the execution of the defensive host cell death program. This seemed a key knowledge gap, as previous work demonstrated that the defensive host cell death was not reliant on a functional type I IFN response [8] and could not be blocked by inhibitors of caspases or mediators of necroptotic cell death [8,9]. However, the CRISPR screen reported in the present study did not uncover genes with known functions in pathogen recognition, immune signaling, or regulated forms of cell death, which likely can be explained by redundancies in the involved host cellular defense and cell death programs. Therefore, we instead focused our attention on other screening hits, which allowed the following two major discoveries, discussed in more depth below. First, CpoS protects *C. trachomatis* from host cellular immune surveillance by stabilizing its parasitophorous vacuole via mediating an adequate sphingolipid supply. Second, CpoS deficiency increases the dihydroceramide to ceramide ratio in infected cells, a metabolic alteration that may contribute to the observed premature death of infected cells.

The CRISPR screen identified several host factors that appear to have specific roles related to CpoS given that deficiencies in these factors protected cells from cytotoxicity following exposure to CTL2-*cpoS*::*cat* but not CTL2 (Fig 1 and S4 Data). We believe that a future in-depth follow-up on the candidates not further investigated in this study, in particular investigations on the roles of the TRAPP complex and TBC1D13, a regulator of RAB35 [42], already previously implicated in CpoS function [11], will be important to obtain a full picture of CpoS’ biological activities. However, in the present study, we decided to focus on the screening hits in the ceramide synthesis pathway. These seemed particularly interesting, as CpoS had previously been revealed to function as a manipulator of membrane trafficking and mediator of sphingolipid transport to the inclusion [8,10–12]. The hits in ceramide synthesis were clearly specific to infections with the *cpoS* mutant. Moreover, they appeared robust given that they included genes mediating not just one but two sequential enzymatic steps in the *de novo* ceramide synthesis pathway, the initiating steps catalyzed by SPT and KDSR, as well as the genes encoding each molecular component of SPT (SPTSSA, SPTLC1, and SPTLC2) (Fig 1 and S4 Data). Notably, while alternative variants of SPT, incorporating components such as SPTSSB and SPTLC3, exist [56], these components are either not or only weakly expressed in HeLa cells according to the Human Protein Atlas [57]. Moreover, there is genetic redundancy in subsequent steps of the *de novo* ceramide synthesis pathway and other ceramide-generating metabolic processes [58], which could explain why enzymes such as ceramide synthases or sphingomyelinases were not uncovered in the screen.

Following up on our hits in ceramide synthesis, we initially pursued the hypothesis that CpoS deficiency may result in a toxic accumulation of sphingolipid metabolites, which may be mitigated by impairments in the *de novo* ceramide synthesis pathway, explaining the cytoprotection achieved in the CRISPR screen. Indeed, in cells infected with CTL2-*cpoS*::*cat*, we observed an accumulation of dihydroceramides and a consequent increase in the dihydroceramide to ceramide ratio (Fig 3 and S5 Fig and S7 Data), a metabolic alteration that is known to have a cytotoxic potential [44] and thus may partake in the premature host cell death. However, it soon became clear that the cytoprotection conferred by disruptions of the ceramide synthesis pathway may not have relied on counteracting this phenomenon alone, as the block in the ceramide synthesis pathway also had profound effects on the ability of CTL2-*cpoS*::*cat* to thrive and replicate in infected cells (Fig 4 and S8 Data). Hence, we followed up on previous leads suggesting CpoS deficiency and SPT inhibition to have inclusion-destabilizing effects [9,51], a task that first required us to develop novel tools for detecting even minor forms of inclusion damage.

Using the influx of host cell cytosolically expressed GFP into the inclusion lumen as an indicator of vacuole integrity, it has previously been shown that inclusion rupture is a natural event during *Chlamydia* exit from host cells, preceding rupture of the host plasma membrane only by minutes [5]. Several *C. trachomatis* mutants, including such deficient for CpoS (CT229/CTL0481), CT383/CTL0639, IncC (CT233/CTL0485), IncM (CT288/CTL0540), IncS (CT147/CTL0402), or GarD (CT135/CTL0390) [9,59–62], as well as certain pharmacological or genetic disturbances, notably including such affecting ceramide synthesis, membrane trafficking, or the cytoskeleton [48,51,63,64], have been proposed to cause inclusions to rupture prematurely, in part in connection with host cell death phenotypes. In the latter studies, inclusion rupture was primarily inferred from the microscopic detection of dispersed intracellular bacteria and/or discontinuous inclusion membrane staining. However, a tool for the specific and sensitive detection of early signs of inclusion instability had been lacking, although such would be crucial to fully resolve questions of causality and the connection between inclusion damage and host cell death. For instance, our previous live cell imaging studies suggested rupture of CpoS-deficient inclusions to occur concurrently with cell death [52]. Hence, it remained unclear if CpoS-deficient inclusions were indeed inherently unstable, *i.e.*, accumulating damage to eventually result in vacuole ruptures, or if the observed ruptures resulted merely as a byproduct of the activation of the host cell death machinery. Similar considerations may apply to other situations in which inclusion rupture has been observed in connection with host cell death.

To monitor vacuole damage during infection with intracellular pathogens, different strategies have been applied, including for instance the detection of damaged vacuole membranes with galectin- or lysenin-based reporters [65–67], and the detection of cytosolically released bacteria, for instance via *uhpT* promoter-driven expression of a fluorescent protein in *Salmonella* [68]. We opted to develop reporters based on the principle of split-GFP [53] – an approach that to our knowledge has not previously been applied in this specific context – because it offers versatility in detecting various signs of damage, including both damaged inclusion membranes and individual bacteria released from inclusions. These novel reporters enabled quantitative analyses of inclusion damage (Figs 5–7 and S9–S11 Data). Moreover, they should be compatible with live cell imaging applications as well, although temporal resolution may be limited by the need for the two parts of GFP to reassemble. Overall, we predict that these reporters will be an important addition to our toolbox for *Chlamydia* spp., as by providing an ability to monitor inclusion stability and the host response to bacteria entering the host cell cytosol, they will also strengthen our ability to mechanistically dissect the host cell-autonomous defense against *Chlamydia* and possibly also enhance our understanding of *Chlamydia* exit from host cells. Moreover, the principle of the approach is simple and should be easily adaptable to other pathogens as well.

Crucially, in the present study, the application of these novel tools revealed that the inclusion damage instigated by the absence of CpoS is indeed subtle initially, characterized by a release of individual bacteria into the host cell cytosol rather than an immediate vacuole rupture (Fig 5). This release of individual bacteria could for instance arise from minor inclusion membrane ruptures followed by membrane resealing. Alternatively, we speculate that inclusion membrane damage may induce membrane repair mechanisms, which may cause a budding of vesicles containing bacteria. These vesicles may subsequently fragment, resulting in the exposure of the bacteria to the host cell cytosol. A rupture of secondary inclusions containing individual bacteria could contribute as well. The detection of these more subtle forms of membrane damage demonstrates that damage in CpoS-deficient inclusions clearly emerges prior to host cell death. Given that cytosolic exposure of bacteria inevitably triggers enhanced activation of immune sensors, this also explains the earlier finding that the absence of CpoS activates the STING-dependent immune signaling pathway in infected cultures hours before the onset of host cell death [8]. While IFN signaling has been shown to induce responses that can destabilize parasitophorous vacuoles [69], a phenomenon previously reported for *Chlamydia* inclusions as well [62,70], our prior finding that the STING pathway-dependent induction of type I IFNs is dispensable for host cell death during infection with the *cpoS* mutant [8], suggests that, in this case, immune signaling may be a response to vacuole damage rather than its cause. What remains insufficiently resolved is the connection between complete vacuole rupture and cell death, *i.e.*, the question if complete rupture precedes cell death induction or is a consequence of the initiation of the cell death process. However, it is noteworthy that artificial, laser-mediated disruption of inclusions has been shown to induce host cell death rapidly [71], which could explain why we typically observe these phenomena only in close connection.

The application of the inclusion damage reporters further allowed us to dissect the critical role of CpoS-mediated sphingolipid acquisition in inclusion stability. It is well-known that *C. trachomatis* can redirect sphingolipid-laden membrane vesicles to the inclusion [47], and we have previously shown that CpoS mediates this transport via its interactions with host Rab GTPases [11]. Hence, our observation that CpoS-deficient inclusions are unstable (Fig 5 and S9 Data) but can be stabilized by the exogenous addition of sphingosine (Fig 7 and S11 Data), a lipid metabolite that is indeed taken up by infected cells and then transported to the inclusion [72], combined with prior findings suggesting that disruptions of membrane trafficking cause inclusions to rupture earlier [48], strongly supports the conclusion that the CpoS-mediated vesicular route of sphingolipid acquisition plays a key role in maintaining inclusion stability.

Interestingly, we observed the inhibition or disruption of the *de novo* ceramide synthesis pathway to destabilize wild-type, CpoS-proficient inclusions only at late infection time points and only in a small proportion of the cells, resulting in correspondingly mild premature cytotoxicity (Figs 2, 6, and S6 and S10 Data). This would suggest the bacteria to be rather independent of *de novo* ceramide synthesis and to primarily utilize the already available lipid pool in the host cell, possibly in combination with exogenous lipids taken up by the cell. While we did not study the effect of CERT depletion on inclusion stability in this study, our findings indicated that CERT depletion also sensitizes cells mildly to CTL2-induced cytotoxicity (S6 Fig and S8 Data), possibly also reflecting a certain degree of inclusion destabilization to occur under these conditions at later infection stages and thus indicating a minor role of the CERT-mediated ceramide transport route in supplying lipids for inclusion stability. Interestingly, a previous study suggested depletion of CERT to have no significant effect on inclusion stability but to strongly reduce *C. trachomatis* growth [48]. The latter phenomenon was not observed in our study but also not investigated in the same manner at the level of inclusion size and progeny formation. It is possible that dependent on the infection conditions, involving different cell types or growth media, the relative importance of the vesicular and non-vesicular sphingolipid supply routes may differ. Moreover, during *in vivo* infection, the dependency on host *de novo* ceramide synthesis may well be modified by the metabolic state of the host cells and the nutrient availability in the infected tissues.

**Fig 8:**
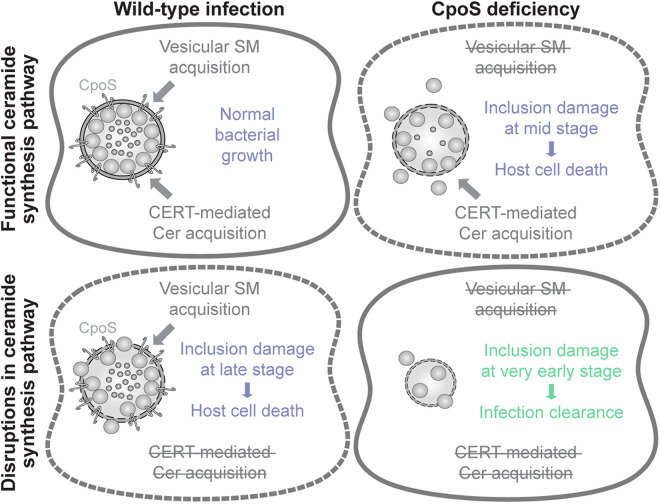
Model describing how a disruption of both sphingolipid supply routes may destabilize inclusions in a potentially therapeutically beneficial manner. *C. trachomatis* can acquire sphingolipids in two ways, by recruiting the host ceramide transfer protein CERT to acquire ceramides (Cer) then converted to sphingomyelin (SM) at the inclusion [48], and by hijacking sphingolipid-laden membrane vesicles and directing them to the inclusion [47]. When CpoS is absent, the vesicular transport of sphingomyelin to the inclusion is compromised [11], leading to inclusion damage at mid stages of infection, followed by premature host cell death. We hypothesize that under conditions of impaired ceramide *de novo* synthesis, the CERT-mediated transport is compromised, while the bacteria may still be able utilize the already available lipid pool through CpoS-mediated modulation of host vesicular transport, leading to more minor inclusion damage that manifests at late stages of infection. A combination of ceramide synthesis disruption and CpoS deficiency, which should compromise both transport routes, results in early inclusion destabilization and clearance of infection while keeping the host cells alive. Hence, an effective therapeutic targeting of inclusion stability may have to aim for blocking both transport routes simultaneously.

Irrespective of the relative contributions of the two distinct sphingolipid supply routes, our finding that CpoS-deficient bacteria are critically dependent on host *de novo* ceramide synthesis (Fig 4 and S8 Data), and possibly more dependent on CERT (S6 Fig and S8 Data), suggests that in situations in which the bacteria cannot satisfy their needs through the modulation of the vesicular route, the non-vesicular route may become essential. Our work further highlighted that a parallel interference with both routes of sphingolipid acquisition, which can be expected to occur when SPT is inhibited during infection with the *cpoS* mutant, destabilizes the vacuoles early on, apparently resulting in infection clearance rather than host cell death (Figs 6 and 8 and S10 Data).

There are several important points future research will need to address. First, the molecular executors of the premature host cell death program activated in the absence of CpoS still need to be identified, and it needs to be clarified if xenophagy contributes to clearing inclusions and cytosolic bacteria when damage occurs early. Second, the vacuole-stabilizing activity of sphingolipids needs to be further dissected mechanistically. It is possible that the stabilization is of mechanical nature, where continued bacterial growth under conditions of impaired sphingolipid acquisition and thus impaired inclusion membrane growth would cause a burst of the inclusion. However, it is also conceivable that a modified lipid composition at the inclusion may influence the recruitment of membrane repair machineries or of vacuole-disrupting immune factors. Third, the possibility that sphingolipids could play a broader role in parasitophorous vacuole stability, for instance, in stabilizing the vacuoles of other host sphingolipid-scavenging intracellular pathogens, such as *Toxoplasma gondii* [73], needs to be investigated. Fourth, the causes for and consequences of the increased dihydroceramide to ceramide ratio in cells infected with the *cpoS* mutant are not fully understood. For instance, it remains to be clarified if this metabolic alteration arises from the altered interactions of the mutant bacteria with the host cellular lipid pool or if it could possibly even reflect the action of a host defense response triggered by the presence of the bacteria in the cytosol. Moreover, considering that an increased dihydroceramide to ceramide ratio was in other instances found to cause cell death by disturbing the integrity of autolysosomal membranes [44], it may even be conceivable that such alteration could by itself contribute to the destabilization of CpoS-deficient inclusions.

Altogether, our work on the *C. trachomatis* effector CpoS further underscores the crucial role of the parasitophorous vacuole as a shelter protecting the pathogen from host cellular immune surveillance, a concept that has now been demonstrated for numerous vacuolar pathogens [74]. Indeed, many intracellular bacteria besides the *Chlamydia* spp. produce effector proteins that partake in the stabilization of their pathogen-containing vacuoles. This includes, for instance, the effectors SifA in *Salmonella* [75] and SdhA in *Legionella* [76]. Collectively, current knowledge suggests that the underlying molecular mechanisms of vacuole stabilization can be diverse and can include but may not be restricted to the modulation of membrane trafficking, membrane repair, cytoskeletal support, and vacuole-targeting immune effectors [74,77]. Experimental conditions that destabilize pathogen vacuoles, such as deficiencies in the above-mentioned virulence factors or processes, seem to generally result in an effective targeting of vacuolar pathogens by the host cell intrinsic defense, typically resulting in the induction of immune signaling and either host cell death or xenophagic clearance, *e.g.*, [76,78–81]. This also suggests that the parasitophorous vacuoles of intracellular pathogens could potentially represent attractive therapeutic targets.

In this broader context, the present work demonstrates the role of the bacterial effector CpoS in preserving the intracellular refuge of *C. trachomatis* by ensuring an adequate supply of sphingolipids. The finding that early inclusion destabilization can eradicate infection without harming the host cells may pave the way for future therapeutic strategies targeting inclusions.

## Materials and methods

### Cell culture

HeLa (ATCC CCL-2), Vero (ATCC CCL-81), and HEK293T (ATCC CRL-3216) cells were cultivated in Dulbecco’s Modified Eagle’s Medium (Gibco), supplemented with 10% heat-inactivated fetal bovine serum (Gibco). In selected experiments, including those involving an analysis of cell death via the resazurin assay, a DMEM medium devoid of phenol red was used to avoid interference with measurements of fluorescence. Cultures were maintained in a humidified incubator (37°C, 5% CO_2_) and routinely tested for contamination with *Mycoplasma* spp. using a commercial PCR-based *Mycoplasma* detection assay (VenorGeM, Minerva).

### Treatment with pharmacological inhibitors or metabolites

The following inhibitors targeting enzymes in ceramide synthesis, metabolism, or transport were used: fumonisin B1 (Cayman), NVP-231 (Cayman), L-cycloserine (Cayman), myriocin (Cayman), D-erythro-MAPP (Cayman), GW4869 (Cayman), SKI-II (BioVision), desipramine hydrochloride (BioVision), and golgicide A (Cayman). Additionally, we used the following sphingolipid metabolites: D-erythro-sphingosine (Merck), D-erythro-dihydrosphingosine (Merck), C6-D-erythro-dihydroceramide (Merck), C6-D-erythro-ceramide (Merck). Stock solutions of inhibitors and metabolites were prepared in DMSO, except for fumonisin B1 and D-erythro-MAPP, which were dissolved in ethanol. The inhibitors and metabolites were added to cell cultures at concentrations and for durations as specified in the respective figure legends.

### *Chlamydia* strains and general infection procedure

Infection experiments were carried out with *C. trachomatis* L2/434/Bu (ATCC VR-902B, CTL2), a variant carrying an insertional disruption of *cpoS* (CTL2-*cpoS*::*cat*), and further genetic derivatives as listed in S1 Table. Two different procedures were used to prepare infection inocula, including crude preparations (used in infection experiments involving the split-GFP system or GFP-expressing bacteria) and highly pure, density-gradient purified EB preparations (used in all other experiments, including the screen). These procedures have been described in detail in a previous study [11]. Bacteria obtained through either of the two methods were resuspended in SPG (sucrose-phosphate-glutamate) buffer (75 g/l sucrose, 0.5 g/l KH_2_PO_4_, 1.2 g/l Na_2_HPO_4_, 0.72 g/l glutamic acid, pH 7.5) and stored at −80°C. Furthermore, the bacterial preparations were titered (as previously described [11]) and confirmed to be free from *Mycoplasma* contamination (as described above for cell lines). To conduct infections, cells were typically seeded in multi-well plates, followed by the addition of bacteria (number of inclusion forming units (IFUs)/cell as specified), centrifugation (1,500 × *g*, 30 min, 23°C), and incubation (37°C, 5% CO_2_) for the indicated periods of time. The centrifugation step was omitted for infections conducted in MatTek dishes. Moreover, infections conducted in the context of the CRISPR screen were done in suspension, as described below.

### CRISPR screen

#### Generation of Cas9-expressing cells.

HeLa cells were lentivirally transduced with pLenti-Cas9-T2A-Blast-BFP (Addgene 196714) to express a codon optimized, wild-type SpCas9, flanked by two nuclear localization signals, and linked to a blasticidin-S-deaminase – mTagBFP fusion protein via a self-cleaving peptide. Following selection with blasticidin, a stable BFP+ population was isolated through repeated sorting for BFP expressors.

#### Guide library construction.

The genome-wide Brunello sgRNA library [82] (which targets 19,114 genes and comprises a total of 77,441 sgRNAs, including about four sgRNAs per gene and 1,000 non-targeting control sgRNAs) was synthesized as 79 bp long oligos (CustomArray, Genscript). The oligo pool was double-stranded by PCR to include an A-U flip in the tracrRNA [83], 10 nucleotide long, untranscribed random sequence labels (RSLs), and an i7 sequencing primer binding site [84]. The PCR product with the sequence ggctttatatat**cttgtggaaaggacgaaacaccgNNNNNNNNNNNNNNNNNNNNgtttaagagctagaaatagcaagtttaaataaggct**agtccgttatcaacttgaaaaagtggcaccgagtcggtgcttttttGATCGGAAGAGCACACGTCTGAACTCCAGTCACNNNNNNNNNNaagcttggcgtaactagatcttgagacaaa (array oligo in bold, i7 primer binding underlined) was cloned by Gibson assembly into pLenti-Puro-AU-flip-3xBsmBI [84] (Addgene 196709). The plasmid library was sequenced to confirm representation and packaged into lentivirus in HEK293T cells using plasmids psPAX2 (Addgene 12260, gift from Didier Trono) and pCMV-VSV-G (Addgene 8454, gift from Bob Weinberg). The virus-containing supernatant was concentrated around 40-fold with Lenti-X concentrator (Takara), aliquoted, and stored in liquid nitrogen.

#### Library virus titration and large-scale transduction.

The functional titer of the library virus was estimated from the fraction of surviving cells after transduction of target cells with different amounts of virus and puromycin selection. For the screen, Cas9-BFP-expressing target cells were transduced in duplicate with the library virus at an approximate multiplicity of infection of 0.3 and a coverage of 1,000 cells per guide in the presence of 2 µg/ml polybrene. Transduced cells were selected with 2 µg/ml puromycin from day 2 to day 4 post transduction, and then frozen at day 6 post transduction and stored at −150°C. Throughout this procedure, the cell count was kept at ≥80 million per replicate to ensure full library coverage.

#### Selection of cells resistant to infection-induced cell death.

Transduced cells were thawed and transferred to large culture flasks for incubation at 37°C, 5% CO_2_. A cell count conducted 12 hrs post-thawing revealed over 90 million live cells. When the cultures approached confluency (4–5 days post-thawing), cells from all flasks were harvested, pooled, and counted. A total of 1 × 10^8^ cells were transferred to a 50 ml tube, washed once with Dulbecco’s phosphate-buffered saline (DPBS), pelleted (800 × *g*, 10 min), and stored at −80°C (=pre-selection cell population). The remaining cells were divided into four batches (each 1.14 × 10^8^ cells) to establish the four sample groups: UI30h, UI60h, KO30h, WT60h. The groups KO30h and WT60h were then infected by addition of 100 µl SPG buffer containing 3.41 x 10^9^ IFUs (30 IFU/cell) of CTL2-*cpoS*::*cat* or CTL2, respectively, while the groups UI30h and UI60h were treated with 100 µl bacteria-free SPG. To enhance infection, the cell suspensions were subjected to two rounds of mixing (by pipetting) and centrifugation (1,500 × *g*, 10 min, 23°C). Subsequently, the cells were transferred to large culture flasks and incubated at 37°C, 5% CO_2_. At the time of cell collection, *i.e.*, at 30 hpi (UI30h and KO30h) or 60 hpi (UI60h and WT60h), the surviving cells were washed twice with DPBS in the flasks (to remove dead cells and cell debris), harvested, counted, washed once with DPBS, pelleted (800 × *g*, 10 min), and stored at −80°C (=selected cell populations). Two independent biological replicates (R1 + R2) of this procedure were conducted, starting from the two independent lentiviral transductions.

#### Isolation of genomic DNA, library preparation, and sgRNA sequencing.

Genomic DNA was isolated from all cell pellets using the QIAamp DNA Blood Maxi Kit (Qiagen). Prior to following the kit’s instructions, RNase A (800 µg/10 million cells) was added to all samples. DNA was quantified using Qubit dsDNA BR Assay Kit (Thermo-Fisher-Scientific). Guide and UMI sequences were amplified by PCR, as previously described [84], using modified primers PCR2_FW (5′-ACACTCTTTCCCTACACGACGCTCTTCCGATCTCTTGTGGAAAGGACGAAACAC-3′) and PCR3_fw (5′-AATGATACGGCGACCACCGAGATCTACAC**[i5]**ACACTCTTTCCCTACACGACGCTCT-3′), respectively. The amplicon was sequenced on Illumina NovaSeq, reading 20 cycles Read 1 with custom primer 5′-CGATCTCTTGTGGAAAGGACGAAACACCG-3′, 10 cycles index read i7 to read the RSL, and six cycles index read i5 to read the sample barcode. The sequencing data were analyzed using the MAGeCK software (v.0.5.6) [85]. RSL counts were not used for data analysis.

### Generation of cell lines with individual gene knockouts or knockdowns

To generate cell lines with individual gene knockouts or knockdowns, the sgRNAs listed in S2 Table were cloned into vector pLentiGuide-Puro (Addgene 52963). Lentiviral particles were harvested from supernatants of HEK293T cells that had been co-transfected with psPAX2 (Addgene 12260), pMD2.G (Addgene 12259), and the respective sgRNA-encoding derivative of pLentiGuide-Puro. After filtration (0.45 µm), virus-containing supernatants were used to transduce Cas9-expressing HeLa cells in the presence of 8–10 µg/ml polybrene (Merck). Cells were co-transduced with the lentiviral vectors encoding the distinct sgRNAs that target the same gene (S2 Table). Transduced cells were selected in presence of puromycin (8–16 µg/ml; Gibco), first added at 72 hrs post transduction. Cells deficient for EXT1 were cloned by limiting dilution. Cells deficient or depleted for other proteins were used as polyclonal cultures.

### Confirmation of gene knockouts or knockdowns by western blot analysis

Protein extracts were prepared by cell lysis in boiling 1% SDS buffer, as previously described [11], separated by SDS PAGE (Mini-PROTEAN TGX 4–20% gels, Bio-Rad), and transferred onto nitrocellulose membranes (pore size of 0.2 µm, Bio-Rad). Membranes were blocked for 20 min with 3% bovine serum albumin (BSA) in Tris-buffered saline with Tween (TBST; 20 mM Tris (pH 7.5), 150 mM NaCl, 0.1% Tween 20) and then incubated overnight at 4°C with primary antibodies diluted in blocking buffer. The following primary antibodies were used: mouse-anti-β-actin (1:2000; Cell Signaling, 3700), rabbit-anti-COG3 (1:2000; Proteintech,11130-1-AP), rabbit-anti-EXT1 (1:1000; Novus, H00002131-D01P), rabbit-anti-KDSR (1:2000; Proteintech, 16228-1-AP), rabbit-anti-SPTLC1 (1:1000; Proteintech, 15376-1-AP), and rabbit-anti-CERT (1:2000; Proteintech, 15191-1-AP). After incubation, membranes were washed thrice with TBST, incubated for one hour with horse radish peroxidase (HRP)-conjugated secondary antibodies (anti-mouse: Thermo-Fisher-Scientific, A10551; anti-rabbit: Thermo-Fisher-Scientific, G-21234; 1:10,000 diluted in blocking buffer), and washed again thrice with TBST. Membranes were then incubated for 1 min with HRP substrate (SuperSignal West Pico PLUS, Thermo-Fisher-Scientific; or ECL Prime Western Blotting Detection Reagent, Cytiva) and chemiluminescent signals were recorded with an Amersham Imager 680RGB (GE Healthcare). Membranes were stripped for 15–30 min with Restore Plus western blot stripping buffer (Thermo-Fisher-Scientific) and blocked with 3% BSA in TBST before detection of additional targets. Based on the results, we considered the cell lines to be either target protein deficient (knockout, KO) when the protein was undetectable, or target protein depleted (knockdown, KD) when reduced protein levels were observed.

### Quantification of surface-exposed HSPG by flow cytometry

For flow cytometric analyses, 2 × 10^5^ cells of each cell line to be analyzed were transferred to a 1.5 ml tube. The cells were washed once with blocking solution (2% BSA in DPBS), then incubated for 30 min with blocking solution and subsequently for 1 hr with primary antibodies (mouse-anti-heparan sulfate, AmsBio, 370255-S, 1:100 diluted in blocking solution). Afterwards, the cells were washed twice with blocking solution, incubated for 30 min with secondary antibodies (goat-anti-mouse-DyLight 650, Thermo-Fisher-Scientific, SA5-10153, 1:400 diluted in blocking solution), and washed again twice. All incubation steps described above were carried out on ice and cells were collected by centrifugation (9,000 × *g*, 5 min, 4°C) between the individual incubation and wash steps. Finally, the cells were resuspended in blocking solution containing propidium iodide (1 µg/ml, Thermo-Fisher-Scientific) prior to measurements at a BD Accuri C6 Plus flow cytometer (BD Biosciences). Data analysis was carried out using FlowJo software (version 10.10.0). Cell debris and doublets were excluded based on forward and side scatter characteristics. Moreover, dead cells were excluded based on propidium iodide fluorescence. Mean DyLight 650 fluorescence intensity was then quantified to assess HSPG surface expression levels.

### Transient and stable expression of GFP1–10 in human cells

Plasmid pcDNA3.1-Cyto-GFP1–10 [54], enabling transient expression of cytosolic GFP1–10, was kindly provided by Kevin Hybiske (University of Washington). HeLa cells were transfected with this plasmid using jetPRIME transfection reagent (Polyplus) with a medium exchange after 2–3 hrs. To enable stable expression of GFP1–10 in HeLa cells, a lentiviral expression system was used. The sequence encoding GFP1–10 was PCR-amplified from pcDNA3.1-Cyto-GFP1–10 using the primers GFP1–10_F (5′-TATGTCGACATGGTTTCGAAAGGCGAGGA-3′) and GFP1–10_R (5′-GGCCTCGAGTTATTTCTCGTTTGGGTCTT-3′). The PCR product was then digested with EcoRI and BamHI and ligated into EcoRI/BamHI-digested lentiviral vector pLVX-Puro (Takara). Subsequently, HEK293T cells were co-transfected with the resulting vector pLVX-Puro-GFP-1–10 and the packaging and envelope plasmids using Lenti-X Packaging Single Shots (Takara). Supernatants containing lentiviral particles were collected at 48 and 72 hrs post-transfection, filtered (0.45 µm), and used to transduce HeLa cells in the presence of 4 µg/ml polybrene (Merck). At 24 hrs post-transduction, the medium was replaced with a medium containing 1 µg/ml puromycin (Gibco) to select for cells that had been stably transduced. Cell clones were generated by limiting dilution and screened for robust GFP1–10 expression. This was based on the fluorescence microscopic detection of GFP signals following infection with strain CTL2/pIncB-GFP11_c_ (described below).

### Generation of GFP11-expressing *Chlamydia* strains

Vector pTL2-tetO-IncB-GFP11x7-FLAG [54], enabling inducible expression of IncB (CT232) tagged at its C-terminus with seven copies of GFP11 (RDHMVLHEYVNAAGIT) and the FLAG epitope (DYKDDDDK), *i.e.*, construct IncB-GFP11_c_, was a kind gift from Kevin Hybiske (University of Washington). Vector pTL2-tetO-CTL0050-GFP11x4-FLAG-CTL0050, enabling inducible expression of OmpA (CTL0050) with four copies of GFP11 (RDHMVLHEYVNAAGIT) and a FLAG epitope (DYKDDDDK) inserted into OmpA’s predicted surface-exposed loop region between the β-strands 11–12, *i.e.*, construct OmpA-GFP11_int_, and vector pTL2-tetO-IncB-GFP11x3-IncB-FLAG, enabling inducible expression of IncB (CTL0484) with a C-terminal FLAG epitope and three copies of GFP11 inserted into IncB’s predicted inclusion lumen-exposed sequence, *i.e.*, construct IncB-GFP11_int_, were generated as follows: Gene blocks encoding the modified OmpA and IncB genes (S1 Methods) were purchased (Thermo-Fisher Scientific). The genes were PCR-amplified (OmpA: 5′-TATAGGTATGCGGCCGCATGAA-3′, 5′-GCGGTAAGTGTCGACTTATTAG-3′; IncB: 5′-ATTATCGGCCGATGGT-3′, 5′-CGCGTGTCGACTTATTAC-3′), digested with NotI and SalI (OmpA) or EagI and SalI (IncB), and inserted into EagI/SalI-digested vector pTL2-tetO-IncB-GFP11x7-FLAG. The vectors encoding the three constructs were then transformed into CTL2 and CTL2-*cpoS*::*cat* using the CaCl_2_ approach [86]. Transformed bacteria were selected in the presence of penicillin G (Merck) and plaque-purified [8]. The presence of the correct plasmid in the clones was verified by PCR (pTL2-F: 5′-TCGATTTTTGTGATGCTCGTCAG-3′, pTL2-R1: 5′-ATATGATCTCTACCTCCAGAGCC-3′) and analysis of PCR products by agarose gel electrophoresis. In experiments using these strains, the expression of GFP11-tagged IncB or OmpA was induced by the addition of 4 ng/ml anhydrotetracycline (Clontech) at 0–2 hpi.

### Fluorometric quantification of host cell viability and bacterial growth

The ability of cells to convert non-fluorescent resazurin into fluorescent resorufin was used as an indicator of cell viability. In brief, resazurin (Merck; 0.15 mg/ml in DPBS) was added to the cells at a volumetric ratio of 1:5, followed by further incubation for 1.5–2.5 hrs (37°C, 5% CO_2_). Resorufin fluorescence was then measured (excitation: 560 nm, emission: 590 nm, bandwidth: 10 nm) and normalized to the fluorescence detected in control cells to calculate cell viability as a percentage. To quantify intracellular bacterial growth fluorometrically, infections were conducted using rsGFP-expressing derivatives of CTL2 and CTL2-*cpoS*::*cat* (S1 Table). At indicated times, the cells were fixed with 4% formaldehyde for 20 min at room temperature. Immediately afterwards, GFP fluorescence was measured (excitation: 490 nm, emission: 510 nm, bandwidth: 10 nm) and normalized to the fluorescence detected in infected control cells to calculate bacterial growth as a percentage. All assays described above were conducted in 96-well plates, and fluorescence measurements were made using a Spark plate reader (Tecan).

### Quantification of sphingolipids

For the quantification of sphingolipids, HeLa cells seeded in 6-well plates were left uninfected or were infected with CTL2, CTL2-*cpoS*::*cat*, or CTL2-*cpoS*::*cat*/pCpoS (10 IFU/cell). In experiments including SKI-II treatment, cells were left uninfected or were infected with CTL2 (5 IFU/cell) in the presence or absence of SKI-II (6.25 µM). At 14 hpi, cell extracts were prepared (from about 1 × 10^6^ cells per sample). In brief, cells were washed with ice-cold DPBS and lysed in ice-cold methanol (HPLC-grade, ≥99.9%, Merck). Lysates were then collected with a cell scraper and stored at −80°C until further processing. Sphingolipids were extracted using 1.5 ml methanol/chloroform (1:1, v:v), containing the internal standards d_7_-dihydrosphingosine (d_7_-dhSph), d_7_-sphingosine (d_7_-Sph), d_7_-sphingosine 1-phosphate (d_7_-S1P), 17:0 ceramide (d18:1/17:0), d_31_-16:0 sphingomyelin (d18:1/16:0-d_31_), 17:0 glucosyl(β) ceramide (d18:1/17:0) and 17:0 lactosyl(β) ceramide (d18:1/17:0) (all from Avanti Polar Lipids) [87]. Final extracts were subjected to sphingolipid quantification by liquid chromatography tandem‐mass spectrometry (LC–MS/MS) applying the multiple reaction monitoring (MRM) approach. Chromatographic separation was achieved on a 1290 Infinity II HPLC (Agilent Technologies) equipped with a Poroshell 120 EC-C8 column (3.0 × 150 mm, 2.7 µm; Agilent Technologies) guarded by a pre-column (3.0 × 5 mm, 2.7 µm) of identical material. MS/MS analyses were carried out using a 6495C triple-quadrupole mass spectrometer (Agilent Technologies) operating in the positive electrospray ionization mode (ESI+). Chromatographic conditions and settings of the ESI source and MS/MS detector have been published elsewhere [88]. The mass transitions used for analysis of sphingolipid subspecies are given in S3 Table. Peak areas of Cer, dhCer, SM, dhSM, HexCer and LacCer subspecies, as determined with MassHunter software (Agilent Technologies), were normalized to those of their internal standards followed by external calibration. DhSph, Sph, and S1P were directly quantified via their deuterated internal standards. Quantification was performed with MassHunter Software (Agilent Technologies).

### Fluorescence microscopy

For standard fluorescence microscopy evaluations (not coupled to electron microscopy), cells were cultivated in 96-well plates or on glass coverslips placed into 24-well plates and fixed for 20 min with 4% formaldehyde in DPBS. Subsequently, the cells were permeabilized for 15 min with 0.2% Triton X-100 in DPBS, incubated for 20 min in blocking solution (2–3% BSA in DPBS), and then for 1 hr in blocking solution containing primary antibodies (rabbit-anti-Slc1 (gift from Raphael Valdivia [89]; 1:1000) or rabbit-anti-FLAG (Merck, F7425; 1:1000)). After washing thrice with DPBS, the cells were incubated for 1 hr in blocking solution containing the DNA dye Hoechst 33342 (Invitrogen; 2−10 µg/ml), Alexa Fluor 555-conjugated secondary antibodies (Invitrogen; 1:250–1:1000), and the whole cell dye HCS CellMask Deep Red Stain (Invitrogen; 5 ng/ml). Subsequently, the cells were washed thrice with DPBS again. Cells grown and stained on glass coverslips were transferred to microscope slides and embedded in ProLong Glass Antifade Mountant (Invitrogen). All incubation steps were conducted at room temperature. In some experiments, staining with the whole cell dye or with antibodies was omitted. Images were captured using various microscopy systems, including an epifluorescence microscope (Zeiss Axio Imager.Z2), a confocal microscope (Leica SP8), and a high-content imaging platform (Molecular Devices ImageXpress Micro Confocal system). Automatic processing of images recorded at the ImageXpress system and detection of chlamydial inclusions and host cell nuclei were performed with CellProfiler (version 4.0.7) [90]. To quantify the extent of inclusion damage, images were analyzed manually. A minimum of 100 cells were analyzed per condition and biological replicate. Cells were classified as “uninfected” or “infected” based on the presence of inclusions. Within the group of “infected” cells, we further differentiated between cells that “contained cytosolic bacteria” and those that “did not contain cytosolic bacteria”.

### FIB-SEM imaging

GFP1–10-expressing HeLa cells were seeded in gridded 35 mm glass bottom MatTek dishes (MatTek, P35G-1.5-14-C-GRD) and subsequently transfected with pcDNA3.1-Cyto-GFP1–10, infected with the indicated strains, and treated with anhydrotetracycline, using above-described standard procedures. At 23.5 hpi, Hoechst 33342 (10 µg/ml) was added to the dishes, and at 24 hpi, the cells were fixed with 4% paraformaldehyde in 0.1 M PHEM buffer. Fluorescence microscopic analysis was conducted using a Leica DMi8 inverted Thunder widefield microscope, equipped with a LEICA DFC 9000 GTC camera and operated by Leica Application Suite X 3.8.2. Subsequently, the cells were postfixed with 2.5% glutaraldehyde in 0.1 M PHEM buffer. Samples were processed for FIB-SEM using a PELCO Biowave Pro+ microwave tissue processor (Ted Pella) following a previously described procedure [91], with minor modifications: calcium was not used during fixation and the contrasting step with lead aspartate was omitted to reduce the risk of overstaining. The samples were embedded in a thin disc of Durcupan resin, mounted on an SEM stub with epoxy and silver glue, and coated with 5 nm platinum. Before imaging, the area of interest was coated with a 1 µm thick protective layer of platinum. The volume was imaged using a FEI Scios DualBeam system, with the acquisition automated using the Auto Slice & View software (ver. 4.1.1.1582). Images were captured with the electron beam operating at 2 kV and 0.2 nA, detected with the T1 in-lens BSE detector. The voxel size (X × Y × Z) of the volume was 9.6 × 9.6 × 20 nm. The volume was further registered and processed in ImageJ using the plugins “Linear Stack Alignment with SIFT” and “MultiStackReg”. After registration, the volume was converted into an MRC file and the header was modified to include pixel size information.

### Statistical analysis

Statistical analyses, other than those described for the CRISPR screen, were conducted in GraphPad Prism 8. The following statistical tests were used when indicated in the figure legends: unpaired *t* test or one-way or two-way ANOVA with the indicated multiple comparison post-hoc tests. The following significance levels were considered: * *p* ≤ 0.05; ** *p* ≤ 0.01; *** *p* ≤ 0.001; ns, not significant. Functional enrichment analysis was conducted  in g:Profiler [13] using the Reactome pathway database [14]. Enrichment maps were drawn using Cytoscape [92,93].

## Supporting information

S1 Fig Overall performance and results of the CRISPR screen. **(A)** Proportion of cells (relative to the respective uninfected controls) that could be recovered at the sampling times (*n* = 2 (R1, R2), mean ± SD). **(B)** Proportion of library sgRNAs detected in the sequenced samples, including Pre-selection (Pre), UI30h, KO30h, UI60h, and WT60h (mean ± SD). **(C)** Distribution of normalized read counts in the indicated samples (median, 5–95 percentile). **(D)** Volcano plots displaying genes with depleted or enriched sgRNAs in cultures infected with CTL2-*cpoS*::*cat* (KO30h versus UI30h) or CTL2 (WT60h versus UI60h). Marked in color, genes depleted or enriched (FC ≤ 0.8 or ≥1.25) with FDR ≤ 0.05 (green) or ≤ 0.2 (lilac). **(E)** Scatter plots displaying genes with sgRNAs found significantly enriched in cultures infected with CTL2-*cpoS*::*cat* (KO30h versus UI30h) or CTL2 (WT60h versus UI60h). Marked in green, hits with FDR ≤ 0.05 in R1 and R2; marked in blue, additional hits with FDR ≤ 0.2 in R1 and R2. Note that all genes marked in green were enriched with FC ≥ 1.25. Numbers mark overlapping dots. The data underlying this figure can be found in S1 and S3 Data.
(TIF)

S2 Fig Functional enrichment analysis of CRISPR screening hits. **(A–B)** Functional enrichment analysis of “general infection hits” and “mutant-specific hits” conducted in g:Profiler using the Reactome pathway database. (A) Bar plots displaying enrichment p-values for all significantly (*p* ≤ 0.05) enriched pathway terms. (B) Cytoscape enrichment maps displaying the relationship between significantly enriched pathway terms. Nodes represent pathways (node size indicates the number of genes in each pathway, node color the significance of the enrichment), and edges their similarity (edge widths indicate the size of similarity). Connected pathways were grouped and genes found enriched are indicated for each group. The data underlying this figure can be found in S4 Data.
(TIF)

S3 Fig Deficiencies in heparan sulfate proteoglycan synthesis provide general protection against *C. trachomatis* L2. **(A)** Schematic overview of the HSPG biosynthetic pathway indicating prominent CRISPR screening hits. HSPG synthesis starts with the attachment of a tetrasaccharide linker to specific serine residues on the core protein, followed by elongation of the sugar chain through addition of disaccharide units. Subsequently, the saccharide residues are modified such as through sulfation, deacetylation, and epimerization. Enzymes encoded by genes identified as screening hits are labeled in bold dark gray (or bold light gray if found with lower confidence). Xyl, xylose; Gal, galactose; GlcA, glucuronic acid; GlcNAc, N-acetylglucosamine; IdoA, iduronic acid; NS, N-sulfation; 2S, 2-O-sulfation; 6S, 6-O-sulfation; 3S, 3-O-sulfation. **(B)** Western blot analysis confirming the absence (knockout, KO) of COG3 and EXT1 in the respective HeLa cell lines. **(C)** Flow cytometric analysis demonstrating reduced levels of cell surface-exposed HSPGs in COG3-deficient cells and EXT1-deficient control cells (mean ± SD, *n* = 3, one-way ANOVA with Dunnett’s post-hoc test; indicated are significant differences compared to the parental (wild-type) cells). **(D)** Fluorescence microscopic analysis demonstrating COG3 deficiency to strongly reduce inclusion formation by CTL2, while having only more moderate effects on infection with CTE. Bacterial inclusions were detected at 28 hpi (mean ± SD, *n* = 3, two-way ANOVA with Sidak’s post-hoc test; if not specified otherwise, indicated are significant differences compared to the parental cells). The data underlying this figure can be found in S5 Data.
(TIF)

S4 Fig Selected inhibitors targeting sphingolipid metabolism protect cells from *cpoS* mutant-induced death. **(A)** The sphingolipid metabolism inhibitors were not cytotoxic at the applied concentrations. Uninfected HeLa cells were treated with the indicated inhibitors at the indicated concentrations. Resorufin fluorescence at 25.5 hrs post treatment is displayed normalized to an untreated control (mean ± SD, *n* = 3, one-way ANOVA with Dunnett’s post-hoc test; no significant differences compared to the untreated control). **(B–C)** MYR, LCS, and GW4869 protected cells from *cpoS* mutant-induced death. HeLa cells were treated with the indicated inhibitors or with solvent only (DMSO) at the indicated concentrations and were parallelly infected with the indicated strains (4 IFU/cell). Resorufin fluorescence (B) and nuclei count (C) at 25.5 hpi are displayed normalized to a CTL2-infected untreated control (mean ± SD, *n* = 7, one-way ANOVA with Dunnett’s post-hoc test; for each strain, indicated are significant differences compared to the DMSO control). The data underlying this figure can be found in S6 Data.
(TIF)

S5 Fig The *cpoS* mutant increases the dihydroceramide to ceramide ratio in infected cells. Quantification of sphingolipid metabolites. Cell extracts from HeLa cells infected with the indicated strains (10 IFU/cell) were prepared at 14 hpi, and the indicated lipids were quantified by LC–MS/MS. Data are represented as heatmaps (means of *n* = 3) indicating log_2_ fold-change (LFC) of metabolite levels or ratios compared to the respective uninfected control (dhCer, dihydroceramides; Cer, ceramides; Sph, sphingosine; S1P, sphingosine-1-phosphate). Selected data are also displayed in Fig 3A. The data underlying this figure can be found in S7 Data.
(TIF)

S6 Fig Effects of membrane trafficking inhibition or CERT depletion on infection with CpoS-proficient and CpoS-deficient strains. **(A)** A blockade in membrane trafficking sensitized CTL2 to the growth-inhibitory action of MYR. HeLa cells were treated with the indicated inhibitors (MYR, 0.5 µM; GCA, 5 µM) and parallelly infected with GFP-expressing derivatives of the indicated strains (2 IFU/cell). Inclusion numbers at 20 hpi are displayed relative to inclusion numbers observed for CTL2 in the absence of inhibitors (mean ± SD, *n* = 2, one-way ANOVA with Tukey’s post-hoc test; if not specified otherwise, for each strain, indicated are significant differences compared to the DMSO control). **(B)** Normalized read counts for CERT-targeting sgRNAs in the CRISPR screen. **(C)** Western blot analysis confirming the depletion (knockdown, KD) of CERT in the respective HeLa cell lines. CERT KD 1 was used for the experiments described in S6D–S6F Fig. **(D)** Depletion of CERT partially protected cells from *cpoS* mutant-induced death. The indicated HeLa cells lines were infected with the indicated strains (5 IFU/cell). Resorufin fluorescence at 24 hpi is displayed normalized to an uninfected control (mean ± SD, *n* = 3, two-way ANOVA with Sidak’s post-hoc test; for each strain, indicated are significant differences compared to the parental cells). **(E–F)** Depletion of CERT did not significantly modify inclusion numbers after infection with the *cpoS* mutant but increased the occurrence of inclusion-free cells. The indicated HeLa cells lines were infected with the indicated strains (5 IFU/cell). (E) Inclusion numbers detected at 24 hpi are displayed normalized to CTL2 inclusion counts in the parental cells (mean ± SD, *n* = 3, two-way ANOVA with Sidak’s post-hoc test; for each strain, indicated are significant differences compared to the parental cells). (F) Representative images showing inclusions (detected by immunofluorescence staining of the bacterial protein Slc1) and DNA (Hoechst) staining (scale = 40 µm). The data underlying this figure can be found in S8 Data.
(TIF)

S7 Fig Fluorescence microscopic tools enabling detection of inclusion damage. **(A)** Fluorescence microscopic validation of the expression and proper localization of the GFP11- and FLAG-tagged constructs in CTL2 and CTL2-*cpoS*::*cat*. HeLa cells, infected with the indicated strains (5 IFU/cell), were fixed, stained (DNA (Hoechst) staining and immunofluorescence detection of FLAG), and imaged at 26 hpi (scale = 20 µm). The “donut-shaped” staining observed for OmpA-GFP11_int_ aligned with its expected localization to the bacterial outer membrane. Furthermore, the patchy localization of IncB-GFP11_C_ and IncB-GFP11_int_ at the inclusion membrane of CTL2 was consistent with the previously reported enrichment of IncB at inclusion membrane microdomains [11]. In cells infected with CTL2-*cpoS*::*cat*, this patchiness was reduced, corroborating our earlier discovery that CpoS deficiency disrupts microdomain formation [11]. **(B)** Fluorescence microscopic detection of split-GFP signals in GFP1–10-expressing HeLa cells infected with the indicated strains of CTL2. HeLa cells were transfected with a plasmid driving GFP1–10 expression, infected with CTL2 (5 IFU/cell), and then fixed, stained (DNA (Hoechst) staining), and imaged at 26 hpi (scale = 20 µm). **(C–D)** Detection of inclusion damage upon treatment with digitonin or DMSO. HeLa cells were transfected with a plasmid driving GFP1–10 expression and infected with the indicated strains of CTL2 (5 IFU/cell). Prior to fixation, DNA (Hoechst) staining, and imaging at 26 hpi, cells were treated with digitonin or DMSO (time and concentration as indicated). A shorter duration of treatment with digitonin (as shown in (D)) preserved the morphology of bacteria in damaged inclusions (scale = 20 µm).
(TIF)

S1 Table *Chlamydia* strains used in this study. (DOCX)

S2 Table sgRNAs used in this study for the generation of individual knockout or knockdown cell lines. (DOCX)

S3 Table LC-MS/MS parameter for sphingolipid quantification. (DOCX)

S1 Methods **Gene blocks used for the cloning of the GFP11-tagged constructs.** Gene blocks used for the generation of vector pTL2-tetO-CTL0050-GFP11x4-FLAG-CTL0050 (pOmpA-GFP11_int_) and pTL2-tetO-IncB-GFP11x3-IncB-FLAG (pIncB-GFP11_int_).
(DOCX)

S1 Raw Images **Raw images of western blots shown in **Figs 2E, S3B**, and **S6C.
(PDF)

S1 Movie FIB-SEM analysis validating inclusion damage at the ultrastructural level. GFP1–10-expressing HeLa cells were infected with CTL2-*cpoS*::*cat*/pOmpA-GFP11_int_ and fixed at 24 hpi. A cell containing green-fluorescent bacteria was identified by fluorescence microscopy and subjected to FIB-SEM analysis. A selected slice of the volume is also depicted in Fig 5E.
(MP4)

S1 Data The data underlying S1A–S1C Fig. **(A)** Proportion of cells that could be recovered at the sampling times. Data are displayed in S1A Fig. **(B)** Proportion of library sgRNAs detected in the sequenced samples. Data are displayed in S1B Fig. **(C)** Distribution of normalized read counts in the indicated samples. Data are displayed in S1C Fig.
(XLSX)

S2 Data Raw results of the analysis of the CRISPR screening data with MAGeCK software. **(A–E)** Results of data analysis for the comparisons UI30h versus pre-selection (A), UI60h versus pre-selection (B), KO30h versus UI30h (C), WT60h versus UI60h (D), and KO30h versus WT60h (E).
(XLSX)

S3 Data The data underlying S1D–S1E Fig. **(A)** Data underlying the volcano plots depicting genes with depleted or enriched sgRNAs in cultures infected with CTL2-*cpoS*::*cat* (KO30h versus UI30h) or CTL2 (WT60h versus UI60h). Data are displayed in S1D Fig. **(B)** Data underlying the scatter plots depicting genes with enriched sgRNAs in cultures infected with CTL2-*cpo*S::*cat* (KO30h versus UI30h) or CTL2 (WT60h versus UI60h). Data are displayed in S1E Fig.
(XLSX)

S4 Data The data underlying Figs 1 and S2. **(A–C)** Data underlying the Venn diagram shown in Fig 1B. (A) Genes identified as “general infection hits”. (B) Genes identified as “mutant-specific hits”. (C) Genes identified as “wildtype-specific hits”. **(D)** Functional enrichment analysis conducted in g:Profiler using the Reactome pathway database. Data are displayed in S2A–S2B Fig. **(E)** Functional categorization of CRISPR screening hits.
(XLSX)

S5 Data The data underlying S3 Fig. **(A)** Flow cytometric measurements of the levels of cell surface-exposed HSPGs in EXT1-deficient and COG3-deficient cells. Data are displayed in S3C Fig. **(B)** Effect of COG3 deficiency on infection with CTL2 and CTE. Data are displayed in S3D Fig.
(XLSX)

S6 Data The data underlying Figs 2 and S4. **(A)** Absence of cytotoxicity by the applied concentrations of the inhibitors of sphingolipid metabolic enzymes. Host cell viability based on resorufin fluorescence measured after 25.5 hrs of treatment with the indicated inhibitors. Data are displayed in S4A Fig. **(B–C)** Selected inhibitors of sphingolipid metabolic enzymes protected cells against *cpoS* mutant-induced cell death. Host cell viability based on resorufin fluorescence (B) and nuclei count (C) measured 25.5 hrs post infection and treatment with the indicated bacterial strains and inhibitors. Data are displayed in S4B Fig (B) and S4C Fig (C). **(D–E)** Selected inhibitors of sphingolipid metabolic enzymes protected cells against *cpoS* mutant-induced cell death and complementation of the mutant restored wild-type phenotypes. Host cell viability based on resorufin fluorescence (D) and nuclei count (E) measured 25.5 hrs post infection and treatment with the indicated bacterial strains and inhibitors. Data are displayed in Fig 2B (D) and Fig 2C (E). **(F–G)** Depletion of SPTLC1 or deficiency in KDSR protected cells against *cpoS* mutant-induced cell death. Host cell viability based on resorufin fluorescence (F) and nuclei count (G) measured 25.5 hrs post infection of the indicated cell lines with the indicated bacterial strains. Data are displayed in Fig 2F (F) and Fig 2G (G).
(XLSX)

S7 Data The data underlying Figs 3 and S5. **(A)** Results from sphingolipidomic analyses demonstrating alterations induced by the *cpoS* mutant. HeLa cells were infected with the indicated strains and lipid extracts were prepared at 14 hpi. Data are displayed in Figs 3A and S5. **(B)** Results from sphingolipidomic analyses testing the effect of SKI-II. Lipid extracts were prepared 14 hrs post infection and treatment. Data are displayed in Fig 3B. **(C)** SKI-II induced death in cells infected with CTL2. Host cell viability based on resorufin fluorescence measured 25.5 hrs post treatment with SKI-II and infection with the indicated bacterial strains. Data are displayed in Fig 3C. **(D)** SKI-II induced death in cells infected with CTL2 at concentrations non-toxic to uninfected cells. Host cell viability based on resorufin fluorescence measured 34 hrs post infection and treatment with the indicated concentrations of SKI-II. Data are displayed in Fig 3D.
(XLSX)

S8 Data The data underlying Figs 4 and S6. **(A)** SKI-II eradicated infected cells from cultures infected with CTL2. Bacterial inclusions were detected by microscopy at 34 hpi. Data are displayed in Fig 4A. **(B)** MYR and LCS, but not GW4869, reduced inclusion count after infection with the *cpoS* mutant. Bacterial inclusions were detected by microscopy at 25.5 hpi. Data are displayed in Fig 4C. **(C)** Differential susceptibility of CTL2 and CTL2-*cpoS*::*cat* to SPT inhibition. GFP fluorescence, as a measure of bacterial growth, was detected at 25.5 hpi. Data are displayed in Fig 4E. **(D)** Depletion of SPTLC1 or deficiency in KDSR partially restored inclusion numbers after infection with the *cpoS* mutant. Bacterial inclusions were detected by microscopy at 24 hpi. Data are displayed in Fig 4F. **(E)** A blockade in membrane trafficking sensitized CTL2 to the growth-inhibitory action of MYR. Bacterial inclusions were detected by microscopy at 20 hpi. Data are displayed in S6A Fig. **(F)** Normalized read counts for CERT-targeting sgRNAs in the CRISPR screen. Data are displayed in S6B Fig. **(G)** Depletion of CERT partially protected cells from *cpoS* mutant-induced death. Host cell viability based on resorufin fluorescence measured 25.5 hrs post infection of the indicated cell lines with the indicated bacterial strains. Data are displayed in S6D Fig. **(H)** Depletion of CERT did not significantly modify inclusion numbers after infection with the *cpoS* mutant. Bacterial inclusions were detected by microscopy at 24 hpi. Data are displayed in S6E Fig.
(XLSX)

S9 Data The data underlying Fig 5. **(A)** Quantitative analysis of inclusion damage in cells infected with the *cpoS* mutant.GFP10-expressing HeLa cells were infected with the indicated strains and then fixed, stained, and imaged at the indicated time points. The percentage of infected cells, surviving cells, and infected cells containing cytosolic bacteria was determined by manual image analysis. Data are displayed in Fig 5F. **(B)** Complementation of the *cpoS* mutant restored inclusion integrity. GFP10-expressing HeLa cells were co-infected with the indicated strains and then fixed, stained, and imaged at 24 hpi. The percentage of infected cells, surviving cells, and infected cells containing cytosolic bacteria was determined by manual image analysis. Data are displayed in Fig 5G.
(XLSX)

S10 Data The data underlying Fig 6. **(A)** Destabilizing effect of SPT inhibition on inclusions. GFP10-expressing HeLa cells were infected with the indicated strains and parallelly treated with MYR or LCS or left untreated (control). Cells were fixed, stained, and imaged at the indicated time points. The percentage of infected cells, surviving cells, and infected cells containing cytosolic bacteria was determined by manual image analysis. Data are displayed in Fig 6A. **(B)** Inclusion-destabilizing effects of SPTLC1 depletion or KDSR deficiency. The indicated cell lines were transfected with a GFP1–10-expressing plasmid and then infected with the indicated strains. Cells were fixed, stained, and imaged at the indicated time points. The percentage of infected cells, surviving cells, and infected cells containing cytosolic bacteria was determined by manual image analysis. Data are displayed in Fig 6C. **(C)** Treatment with SKI-II destabilizes CTL2 inclusions. GFP10-expressing HeLa cells were infected with the indicated strains and parallelly treated with SKI-II or solvent-only (DMSO). Cells were fixed at 24 hpi, stained, and imaged. The percentage of infected cells displaying inclusion damage was determined by manual image analysis. Data are displayed in Fig 6D.
(XLSX)

S11 Data The data underlying Fig 7. **(A)** Supplementation of growth media with sphingoid bases protected cells from *cpoS* mutant-induced death. Host cell viability based on nuclei count was measured 25.5 hrs post infection and treatment with the indicated bacterial strains and metabolites. Data are displayed in Fig 7A. **(B)** Supplementation of growth media with sphingoid bases restored the growth of the *cpoS* mutant. Bacterial growth based on inclusion count was measured 25.5 hrs post infection and treatment with the indicated bacterial strains and metabolites. Data are displayed in Fig 7B. **(C)** Stabilizing effect of sphingosine on CpoS-deficient inclusions. GFP10-expressing HeLa cells were infected with the indicated strains and parallelly treated with 5 µM sphingosine (Sph) or left untreated (control). Cells were fixed, stained, and imaged at 24 hpi. The percentage of infected cells, surviving cells, and infected cells containing cytosolic bacteria was determined by manual image analysis. Data are displayed in Fig 7C.
(XLSX)

## Abbreviations

**:**
Cer: ceramide
COG complex: conserved oligomeric Golgi complex
dhCer: dihydroceramide
dhSph: dihydrosphingosine
EB: elementary body
FC: fold change
FDR: false discovery rate
GARP complex: Golgi-associated retrograde protein complex
GFP: green-fluorescent protein
hpi: hours post-infection
HSPG: heparan sulfate proteoglycan
Inc: inclusion membrane protein
IFN: interferon
IFU: inclusion-forming unit
LCS: L-cycloserine
MYR: myriocin
RB: reticulate body
SM: sphingomyelin
Sph: sphingosine
SPT: serine palmitoyltransferase
