# Supplementary figures and images for: Genome-wide identification of modulators of Chlamydia trachomatis parasitophorous vacuole stability highlights an important role for sphingolipid supply
Source: PLoS Biol. 2025 Aug 12;23(8):e3003297. doi: 10.1371/journal.pbio.3003297 (PMC12342332; doi:10.1371/journal.pbio.3003297)

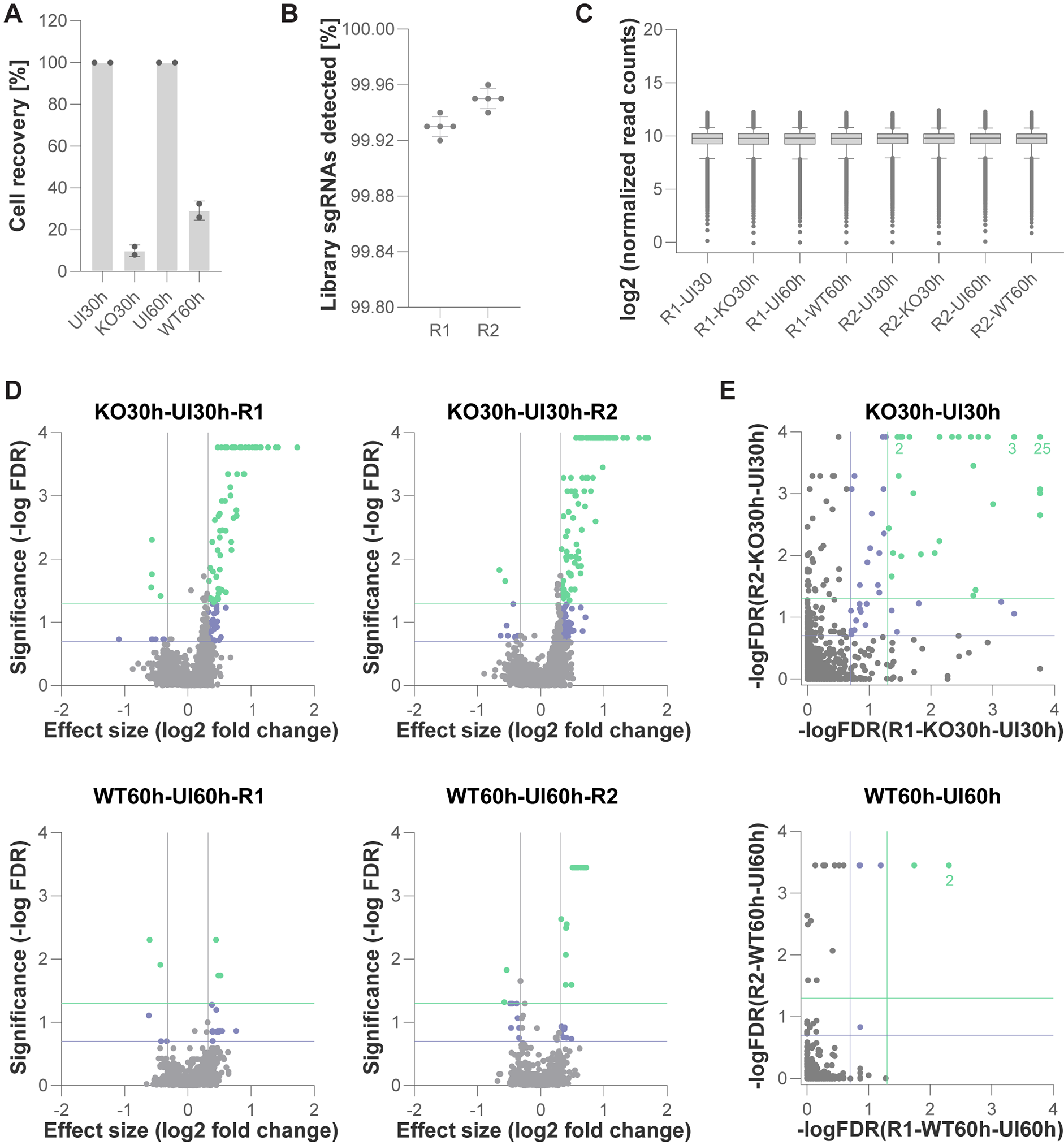

Supplement: S1 Fig — (A) Proportion of cells (relative to the respective uninfected controls) that could be recovered at the sampling times (n = 2 (R1, R2), mean ± SD). (B) Proportion of library sgRNAs detected in the sequenced samples, including Pre-selection (Pre), UI30h, KO30h, UI60h, and WT60h (mean ± SD). (C) Distribution of normalized read counts in the indicated samples (median, 5–95 percentile). (D) Volcano plots displaying genes with depleted or enriched sgRNAs in cultures infected with CTL2-cpoS::cat (KO30h versus UI30h) or CTL2 (WT60h versus UI60h). Marked in color, genes depleted or enriched (FC ≤ 0.8 or ≥1.25) with FDR ≤ 0.05 (green) or ≤ 0.2 (lilac). (E) Scatter plots displaying genes with sgRNAs found significantly enriched in cultures infected with CTL2-cpoS::cat (KO30h versus UI30h) or CTL2 (WT60h versus UI60h). Marked in green, hits with FDR ≤ 0.05 in R1 and R2; marked in blue, additional hits with FDR ≤ 0.2 in R1 and R2. Note that all genes marked in green were enriched with FC ≥ 1.25. Numbers mark overlapping dots. The data underlying this figure can be found in S1 and S3 Data. (TIF) [file pbio.3003297.s001.tif]

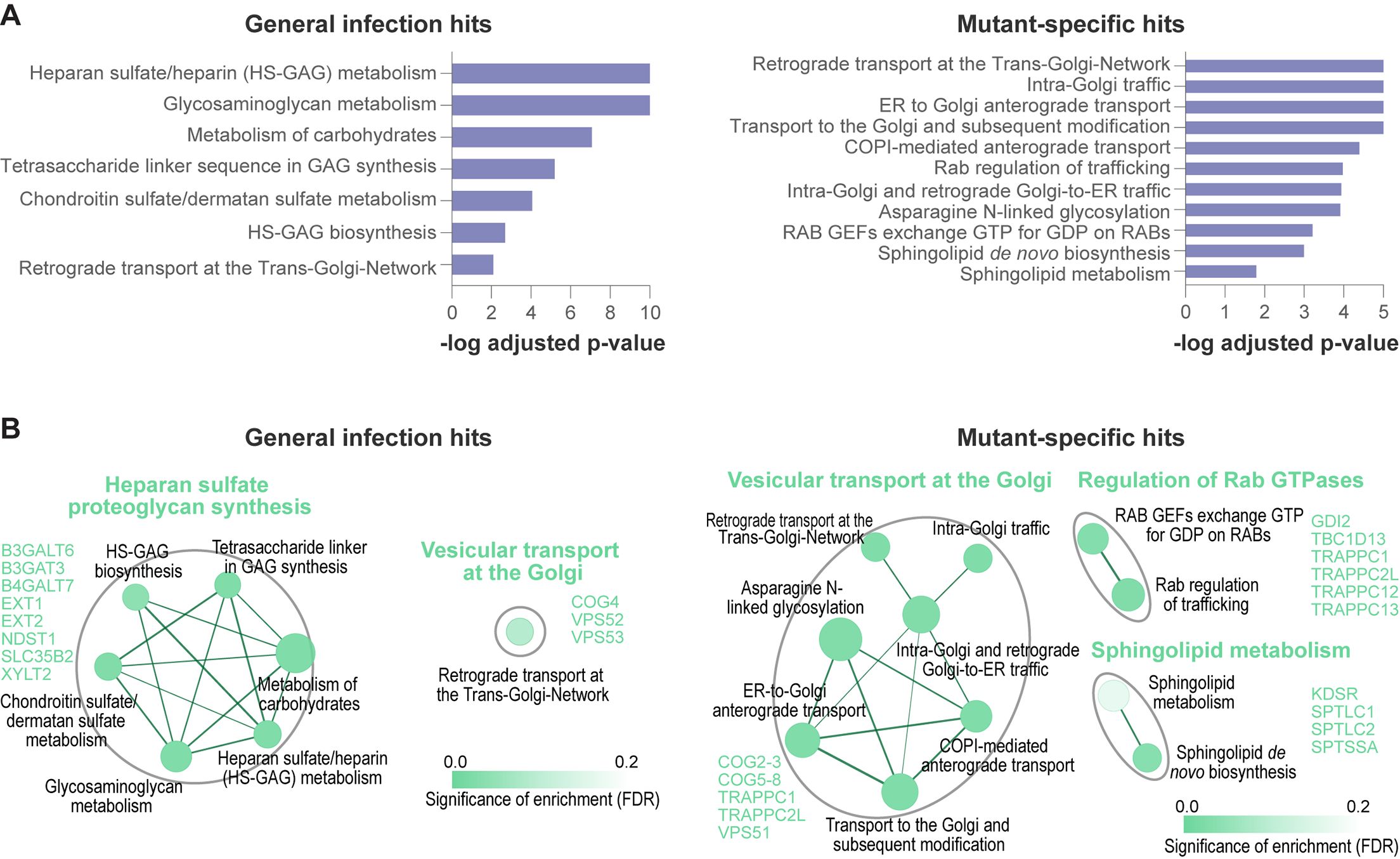

Supplement: S2 Fig — (A–B) Functional enrichment analysis of “general infection hits” and “mutant-specific hits” conducted in g:Profiler using the Reactome pathway database. (A) Bar plots displaying enrichment p-values for all significantly (p ≤ 0.05) enriched pathway terms. (B) Cytoscape enrichment maps displaying the relationship between significantly enriched pathway terms. Nodes represent pathways (node size indicates the number of genes in each pathway, node color the significance of the enrichment), and edges their similarity (edge widths indicate the size of similarity). Connected pathways were grouped and genes found enriched are indicated for each group. The data underlying this figure can be found in S4 Data. (TIF) [file pbio.3003297.s002.tif]

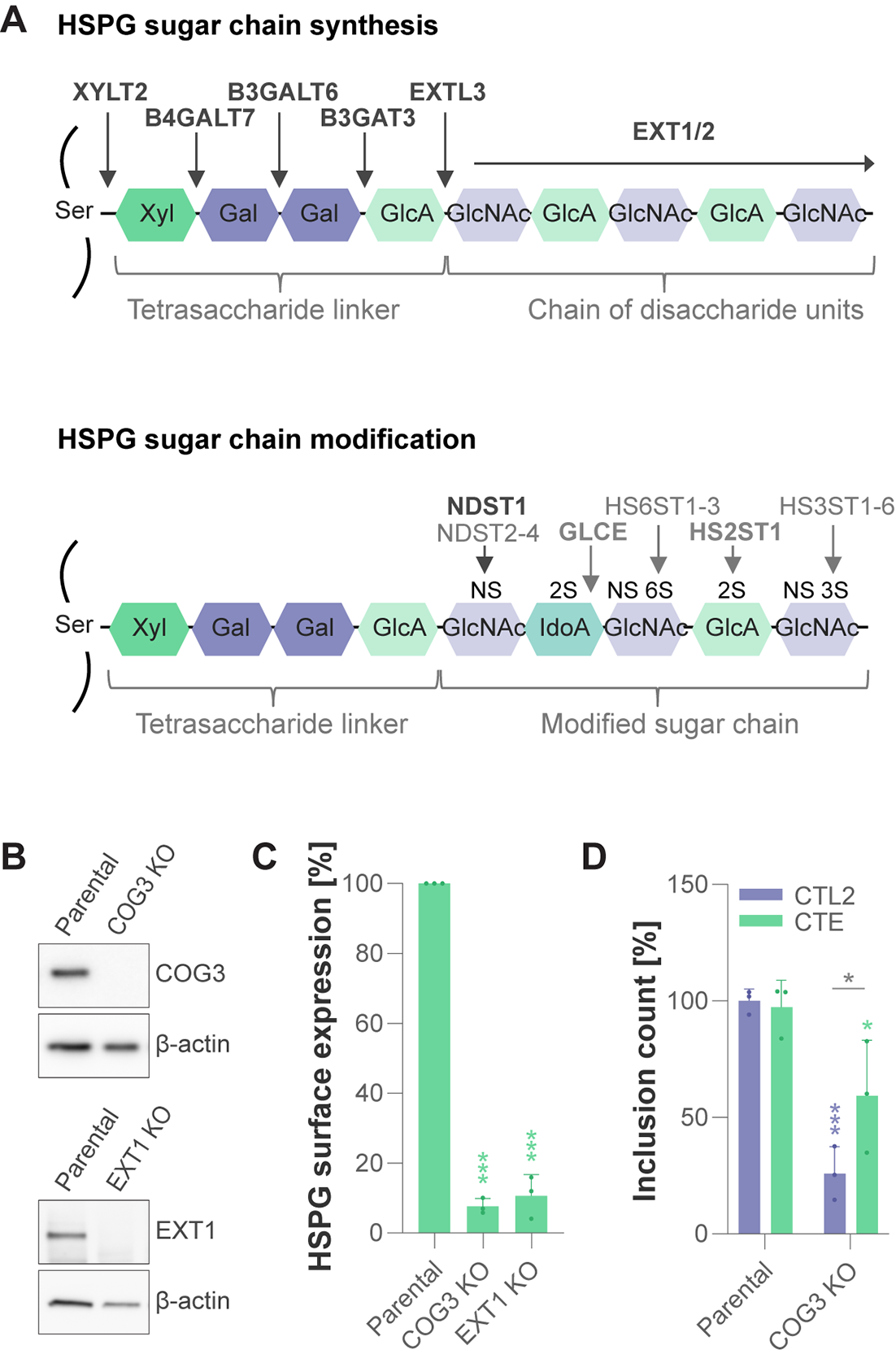

Supplement: S3 Fig — (A) Schematic overview of the HSPG biosynthetic pathway indicating prominent CRISPR screening hits. HSPG synthesis starts with the attachment of a tetrasaccharide linker to specific serine residues on the core protein, followed by elongation of the sugar chain through addition of disaccharide units. Subsequently, the saccharide residues are modified such as through sulfation, deacetylation, and epimerization. Enzymes encoded by genes identified as screening hits are labeled in bold dark gray (or bold light gray if found with lower confidence). Xyl, xylose; Gal, galactose; GlcA, glucuronic acid; GlcNAc, N-acetylglucosamine; IdoA, iduronic acid; NS, N-sulfation; 2S, 2-O-sulfation; 6S, 6-O-sulfation; 3S, 3-O-sulfation. (B) Western blot analysis confirming the absence (knockout, KO) of COG3 and EXT1 in the respective HeLa cell lines. (C) Flow cytometric analysis demonstrating reduced levels of cell surface-exposed HSPGs in COG3-deficient cells and EXT1-deficient control cells (mean ± SD, n = 3, one-way ANOVA with Dunnett’s post-hoc test; indicated are significant differences compared to the parental (wild-type) cells). (D) Fluorescence microscopic analysis demonstrating COG3 deficiency to strongly reduce inclusion formation by CTL2, while having only more moderate effects on infection with CTE. Bacterial inclusions were detected at 28 hpi (mean ± SD, n = 3, two-way ANOVA with Sidak’s post-hoc test; if not specified otherwise, indicated are significant differences compared to the parental cells). The data underlying this figure can be found in S5 Data. (TIF) [file pbio.3003297.s003.tif]

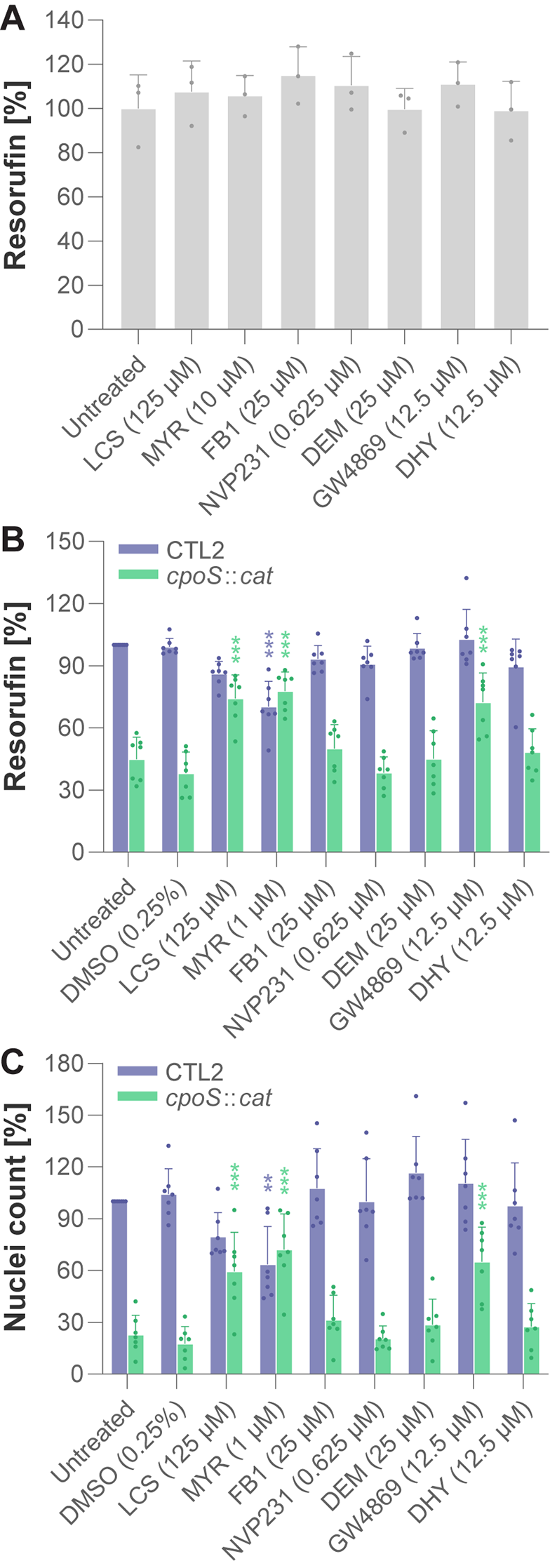

Supplement: S4 Fig — (A) The sphingolipid metabolism inhibitors were not cytotoxic at the applied concentrations. Uninfected HeLa cells were treated with the indicated inhibitors at the indicated concentrations. Resorufin fluorescence at 25.5 hrs post treatment is displayed normalized to an untreated control (mean ± SD, n = 3, one-way ANOVA with Dunnett’s post-hoc test; no significant differences compared to the untreated control). (B–C) MYR, LCS, and GW4869 protected cells from cpoS mutant-induced death. HeLa cells were treated with the indicated inhibitors or with solvent only (DMSO) at the indicated concentrations and were parallelly infected with the indicated strains (4 IFU/cell). Resorufin fluorescence (B) and nuclei count (C) at 25.5 hpi are displayed normalized to a CTL2-infected untreated control (mean ± SD, n = 7, one-way ANOVA with Dunnett’s post-hoc test; for each strain, indicated are significant differences compared to the DMSO control). The data underlying this figure can be found in S6 Data. (TIF) [file pbio.3003297.s004.tif]

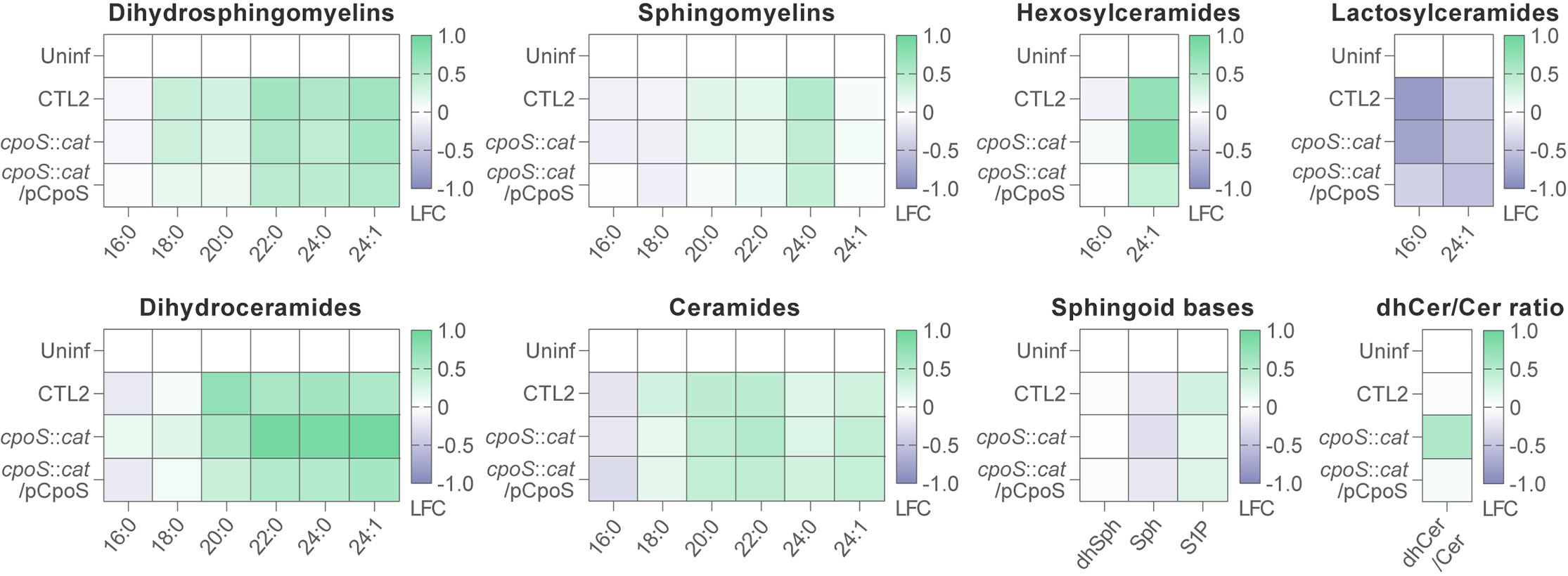

Supplement: S5 Fig — Quantification of sphingolipid metabolites. Cell extracts from HeLa cells infected with the indicated strains (10 IFU/cell) were prepared at 14 hpi, and the indicated lipids were quantified by LC–MS/MS. Data are represented as heatmaps (means of n = 3) indicating log2 fold-change (LFC) of metabolite levels or ratios compared to the respective uninfected control (dhCer, dihydroceramides; Cer, ceramides; Sph, sphingosine; S1P, sphingosine-1-phosphate). Selected data are also displayed in Fig 3A. The data underlying this figure can be found in S7 Data. (TIF) [file pbio.3003297.s005.tif]

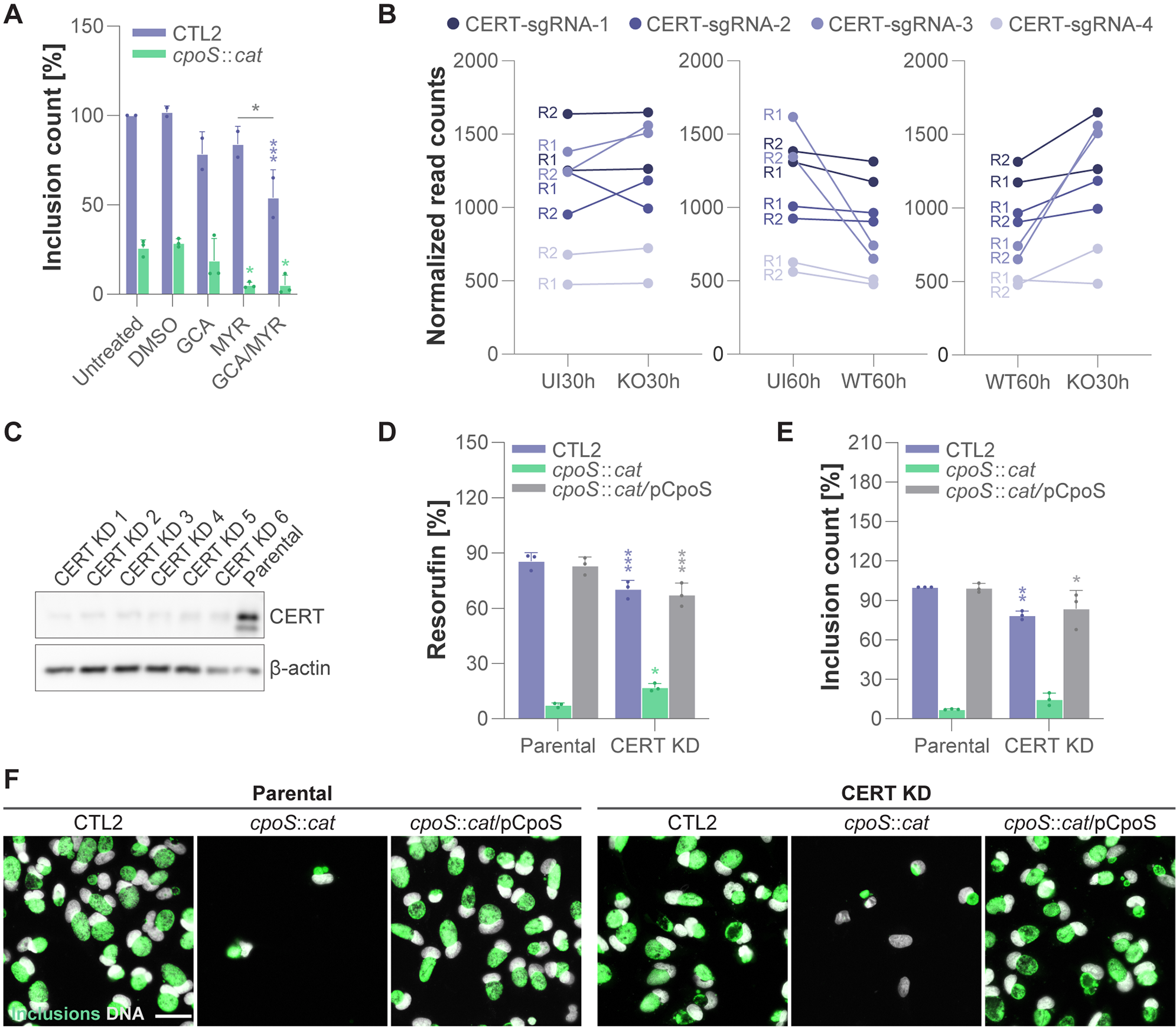

Supplement: S6 Fig — (A) A blockade in membrane trafficking sensitized CTL2 to the growth-inhibitory action of MYR. HeLa cells were treated with the indicated inhibitors (MYR, 0.5 µM; GCA, 5 µM) and parallelly infected with GFP-expressing derivatives of the indicated strains (2 IFU/cell). Inclusion numbers at 20 hpi are displayed relative to inclusion numbers observed for CTL2 in the absence of inhibitors (mean ± SD, n = 2, one-way ANOVA with Tukey’s post-hoc test; if not specified otherwise, for each strain, indicated are significant differences compared to the DMSO control). (B) Normalized read counts for CERT-targeting sgRNAs in the CRISPR screen. (C) Western blot analysis confirming the depletion (knockdown, KD) of CERT in the respective HeLa cell lines. CERT KD 1 was used for the experiments described in S6D–S6F Fig. (D) Depletion of CERT partially protected cells from cpoS mutant-induced death. The indicated HeLa cells lines were infected with the indicated strains (5 IFU/cell). Resorufin fluorescence at 24 hpi is displayed normalized to an uninfected control (mean ± SD, n = 3, two-way ANOVA with Sidak’s post-hoc test; for each strain, indicated are significant differences compared to the parental cells). (E–F) Depletion of CERT did not significantly modify inclusion numbers after infection with the cpoS mutant but increased the occurrence of inclusion-free cells. The indicated HeLa cells lines were infected with the indicated strains (5 IFU/cell). (E) Inclusion numbers detected at 24 hpi are displayed normalized to CTL2 inclusion counts in the parental cells (mean ± SD, n = 3, two-way ANOVA with Sidak’s post-hoc test; for each strain, indicated are significant differences compared to the parental cells). (F) Representative images showing inclusions (detected by immunofluorescence staining of the bacterial protein Slc1) and DNA (Hoechst) staining (scale = 40 µm). The data underlying this figure can be found in S8 Data. (TIF) [file pbio.3003297.s006.tif]

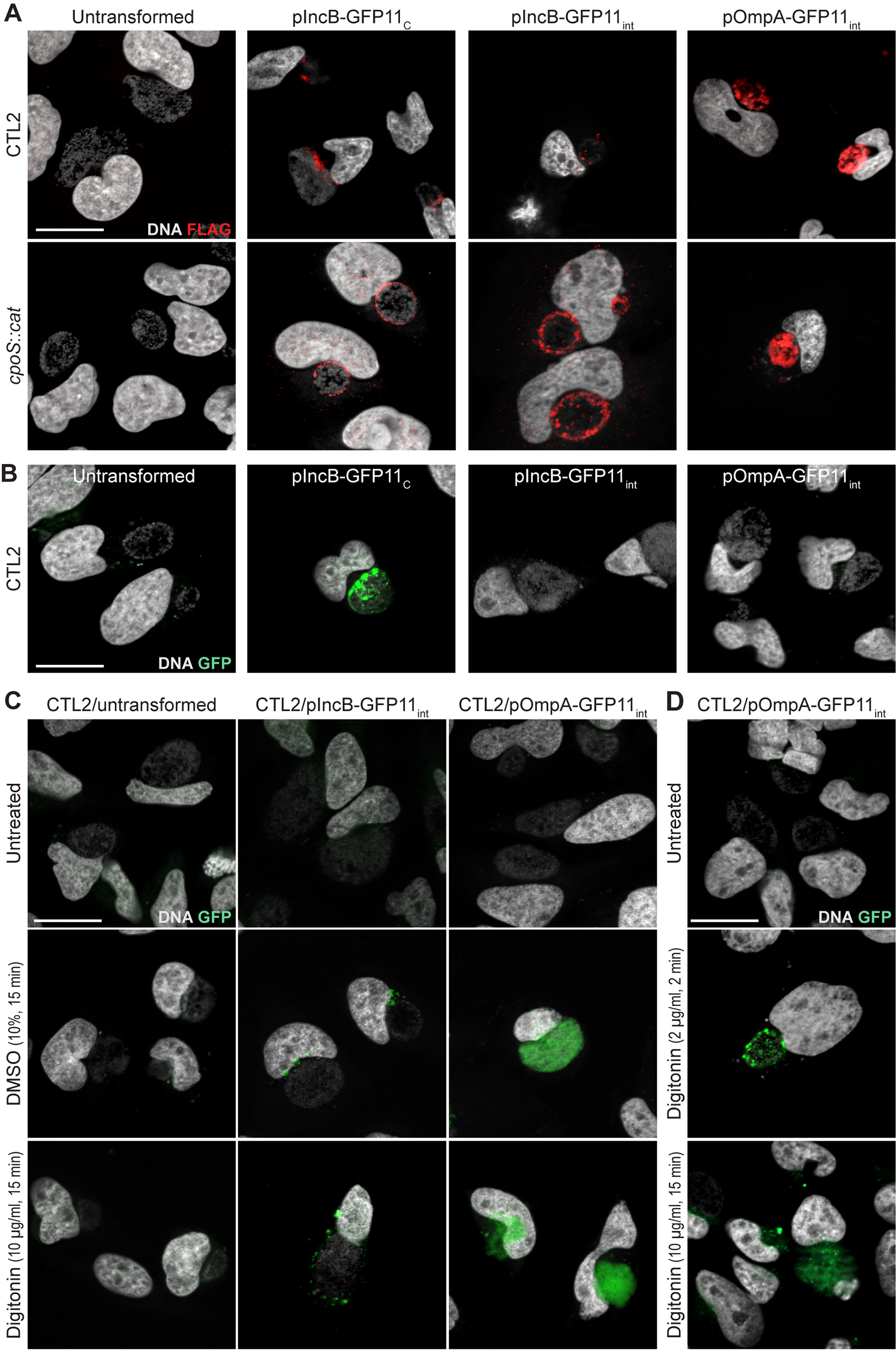

Supplement: S7 Fig — (A) Fluorescence microscopic validation of the expression and proper localization of the GFP11- and FLAG-tagged constructs in CTL2 and CTL2-cpoS::cat. HeLa cells, infected with the indicated strains (5 IFU/cell), were fixed, stained (DNA (Hoechst) staining and immunofluorescence detection of FLAG), and imaged at 26 hpi (scale = 20 µm). The “donut-shaped” staining observed for OmpA-GFP11int aligned with its expected localization to the bacterial outer membrane. Furthermore, the patchy localization of IncB-GFP11C and IncB-GFP11int at the inclusion membrane of CTL2 was consistent with the previously reported enrichment of IncB at inclusion membrane microdomains [11]. In cells infected with CTL2-cpoS::cat, this patchiness was reduced, corroborating our earlier discovery that CpoS deficiency disrupts microdomain formation [11]. (B) Fluorescence microscopic detection of split-GFP signals in GFP1–10-expressing HeLa cells infected with the indicated strains of CTL2. HeLa cells were transfected with a plasmid driving GFP1–10 expression, infected with CTL2 (5 IFU/cell), and then fixed, stained (DNA (Hoechst) staining), and imaged at 26 hpi (scale = 20 µm). (C–D) Detection of inclusion damage upon treatment with digitonin or DMSO. HeLa cells were transfected with a plasmid driving GFP1–10 expression and infected with the indicated strains of CTL2 (5 IFU/cell). Prior to fixation, DNA (Hoechst) staining, and imaging at 26 hpi, cells were treated with digitonin or DMSO (time and concentration as indicated). A shorter duration of treatment with digitonin (as shown in (D)) preserved the morphology of bacteria in damaged inclusions (scale = 20 µm). (TIF) [file pbio.3003297.s007.tif]
