# Supplementary material for: Genome-wide identification of modulators of Chlamydia trachomatis parasitophorous vacuole stability highlights an important role for sphingolipid supply
Source: PLoS Biol. 2025 Aug 12;23(8):e3003297. doi: 10.1371/journal.pbio.3003297 (PMC12342332; doi:10.1371/journal.pbio.3003297)
Supplement: S1 Table — (DOCX) [file pbio.3003297.s008.docx]

**S1 Table. *Chlamydia* strains used in this study.**

| **Strain name** | **Short names**  **(used in this study)** | **Description** | **Source** |
| --- | --- | --- | --- |
|  | | | |
| **Wild-type strain** | | | |
| CTL2 | CTL2 | *Chlamydia trachomatis* strain L2/434/Bu | ATCC VR-902B |
|  | | | |
| **CpoS-deficient and complemented strains** | | | |
| CTL2-*cpoS*::*cat* | CTL2-*cpoS*::*cat*  (or: *cpoS*::*cat*) | Derived from CTL2, *cat* insertion in *cpoS*, expresses non-functional CpoS truncated after amino acid 92 | Meier et al 2023 ^1^ |
| CTL2-*cpoS*::*cat* / pCpoS(L2)-FLAG | CTL2-*cpoS*::*cat* /pCpoS  (or: *cpoS*::*cat* /pCpoS) | Derived from CTL2-*cpoS*::*cat*, expresses CTL2 CpoS C-terminally tagged with a FLAG tag (constitutive) |  |
|  | | | |
| **GFP-expressing strains** | | | |
| CTL2 / p2TK2-SW2-rsGFP | CTL2 /pGFP | Derived from CTL2, expresses rsGFP (constitutive) | Generated, plasmid from Agaisse and Derré 2013 ^2^ |
| CTL2-*cpoS*::*cat* / p2TK2-SW2-rsGFP | CTL2-*cpoS*::*cat* /pGFP  (or: *cpoS*::*cat* /pGFP) | Derived from CTL2-*cpoS*::*cat*, expresses rsGFP (constitutive) |  |
|  | | | |
| **GFP11-expressing strains** | | | |
| CTL2 / pTL2-tetO-IncB-GFP11x7-FLAG | CTL2 /pIncB-GFP11_c_ | Derived from CTL2, expresses IncB C-terminally tagged with seven repeats of GFP11 and a FLAG tag (anhydrotetracycline-inducible) | Generated, plasmid from Wang et al 2018 ^3^ |
| CTL2-*cpoS*::*cat* / pTL2-tetO-IncB-GFP11x7-FLAG | CTL2-*cpoS*::*cat* /pIncB-GFP11_c_ | Derived from CTL2-*cpoS*::*cat*, expresses IncB C-terminally tagged with seven repeats of GFP11 and a FLAG tag (anhydrotetracycline-inducible) |  |
| CTL2 / pTL2-tetO-IncB-GFP11x3-IncB-FLAG | CTL2 /pIncB-GFP11_int_ | Derived from CTL2, expresses IncB containing three internal repeats of GFP11 and a C-terminal FLAG tag (anhydrotetracycline-inducible) | Generated with plasmids developed in this study |
| CTL2-*cpoS*::*cat* / pTL2-tetO-IncB-GFP11x3-IncB-FLAG | CTL2-*cpoS*::*cat* /pIncB-GFP11_int_ | Derived from CTL2-*cpoS*::*cat*, expresses IncB containing three internal repeats of GFP11 and a C-terminal FLAG tag (anhydrotetracycline-inducible) |  |
| CTL2 / pTL2-tetO-CTL0050-GFP11x4-FLAG-CTL0050 | CTL2 /pOmpA-GFP11_int_ | Derived from CTL2, expresses OmpA containing four internal repeats of GFP11 and a FLAG tag between OmpA’s β-strands 11 and 12 (anhydrotetracycline-inducible) |  |
| CTL2-*cpoS*::*cat* / pTL2-tetO-CTL0050-GFP11x4-FLAG-CTL0050 | CTL2-*cpoS*::*cat* /pOmpA-GFP11_int_ | Derived from CTL2-*cpoS*::*cat*, expresses OmpA containing four internal repeats of GFP11 and a FLAG tag between OmpA’s β-strands 11 and 12 (anhydrotetracycline-inducible) |  |

1. Meier, K., Jachmann, L.H., Türköz, G., Babu Sait, M.R., Pérez, L., Kepp, O., Valdivia, R.H., Kroemer, G., and Sixt, B.S. (2023). The *Chlamydia* effector CpoS modulates the inclusion microenvironment and restricts the interferon response by acting on Rab35. mBio *14*, e0319022.

2. Agaisse, H., and Derre, I. (2013). A *C. trachomatis* cloning vector and the generation of *C. trachomatis* strains expressing fluorescent proteins under the control of a *C. trachomatis* promoter. PLoS One *8*, e57090. 10.1371/journal.pone.0057090.

3. Wang, X., Hybiske, K., and Stephens, R.S. (2018). Direct visualization of the expression and localization of chlamydial effector proteins within infected host cells. Pathog Dis *76*, fty011. 10.1093/femspd/fty011.

**References**
