# Supplementary material for: Genome-wide identification of modulators of Chlamydia trachomatis parasitophorous vacuole stability highlights an important role for sphingolipid supply
Source: PLoS Biol. 2025 Aug 12;23(8):e3003297. doi: 10.1371/journal.pbio.3003297 (PMC12342332; doi:10.1371/journal.pbio.3003297)
Supplement: S2 Table — (DOCX) [file pbio.3003297.s009.docx]

**S2 Table. sgRNAs used in this study for the generation of individual knockout or knockdown cell lines.**

| **Target gene** | **sgRNAs (5’-3’)** |
| --- | --- |
| COG3 | TCACTATTCACCGACAATGT, TTATTGAACAAATAGAACTG, ACATTGTCGGTGAATAGTGA |
| EXT1 | CAATCTCTCATCGCCTATGA, GGATGATCCTTAGAAAAGAG, GATTGTATTAACTACACTAG |
| SPTLC1 | TCCTGCTTACCATCTTATTT |
| KDSR | AATGCACTTCCCGATGCCAC, GACATGCTGGTAAATTGTGC |
| CERT | TGAACTAATGGTTAAACGTG |
