# Supplementary material for: Genome-wide identification of modulators of Chlamydia trachomatis parasitophorous vacuole stability highlights an important role for sphingolipid supply
Source: PLoS Biol. 2025 Aug 12;23(8):e3003297. doi: 10.1371/journal.pbio.3003297 (PMC12342332; doi:10.1371/journal.pbio.3003297)
Supplement: S3 Table — (DOCX) [file pbio.3003297.s010.docx]

**S3 Table. LC-MS/MS parameter for sphingolipid quantification.**

| **Sphingolipid ^a^** | **Precursor ion (m/z)** | **Product ion**  **(m/z) ^b^** | **Retention time (min)** | **Internal Standards ^a^** |
| --- | --- | --- | --- | --- |
| d_7_-Sph | 307.3 [M+H]^+^ | **289.3 (8)** / 259.3 (20) | 5.5 | - |
| d_7_-dhSph | 309.4 [M+H]^+^ | **291.3 (12)** / 261.3 (24) | 5.8 | - |
| d_7_-S1P | 387.3 [M+H]^+^ | **271.3 (20)** / 82.1 (36) | 6.8 | - |
| Sph | 300.3 [M+H]^+^ | **282.3 (8)** / 252.3 (16) | 5.5 | d_7_-Sph |
| dhSph | 302.3 [M+H]^+^ | **284.3 (12)** / 254.3 (20) | 5.8 | d_7_-dhSph |
| S1P | 380.3 [M+H]^+^ | **264.3 (20)** / 82.1 (32) | 6.8 | d_7_-S1P |
| 16:0 dhCer | 540.5 [M+H]^+^ | **522.6 (20)** / 284.3 (28) | 14.2 | 17:0 Cer |
| 18:0 dhCer | 568.5 [M+H]^+^ | **550.5 (20)** / 284.3 (28) | 16.3 | 17:0 Cer |
| 20:0 dhCer | 596.6 [M+H]^+^ | **578.6 (22)** / 284.3 (32) | 18.9 | 17:0 Cer |
| 22:0 dhCer | 624.6 [M+H]^+^ | **606.6 (22)** / 284.3 (32) | 22.1 | 17:0 Cer |
| 24:0 dhCer | 652.7 [M+H]^+^ | **634.6 (24)** / 284.3 (36) | 25.7 | 17:0 Cer |
| 24:1 dhCer | 650.7 [M+H]^+^ | **632.7 (24)** / 284.3 (36) | 22.6 | 17:0 Cer |
| 17:0 Cer | 534.5 [M-H_2_O+H]^+^ | **264.3 (24)** / 282.3 (28) | 14.6 | - |
| 16:0 Cer | 520.5 [M-H_2_O+H]^+^ | **264.3 (24)** / 282.3 (24) | 13.7 | 17:0 Cer |
| 18:0 Cer | 548.5 [M-H_2_O+H]^+^ | **264.2 (24)** / 282.3 (28) | 15.6 | 17:0 Cer |
| 20:0 Cer | 576.6 [M-H_2_O+H]^+^ | **264.3 (32)** / 282.3 (28) | 18 | 17:0 Cer |
| 22:0 Cer | 604.6 [M-H_2_O+H]^+^ | **264.3 (34)** / 282.3 (30) | 21 | 17:0 Cer |
| 24:0 Cer | 632.6 [M-H_2_O+H]^+^ | **264.3 (36)** / 282.3 (28) | 24.5 | 17:0 Cer |
| 24:1 Cer | 630.6 [M-H_2_O+H]^+^ | **264.3 (36)** / 282.3 (32) | 21.2 | 17:0 Cer |
| 16:0 dhSM | 705.6 [M+H]^+^ | **184.0 (8)** / 86.1 (76) | 13.5 | d_31_-16:0 SM |
| 18:0 dhSM | 733.6 [M+H]^+^ | **184.0 (28)** / 86.1 (76) | 15.6 | d_31_-16:0 SM |
| 20:0 dhSM | 761.6 [M+H]^+^ | **184.0 (28)** / 86.1 (78) | 18 | d_31_-16:0 SM |
| 22:0 dhSM | 789.7 [M+H]^+^ | **184.0 (28)** / 86.1 (78) | 20.9 | d_31_-16:0 SM |
| 24:0 dhSM | 817.7 [M+H]^+^ | **184.0 (28)** / 86.1 (80) | 24.5 | d_31_-16:0 SM |
| 24:1 dhSM | 815.7 [M+H]^+^ | 184.0 (8) **/ 86.1 (80)** | 20.9 | d_31_-16:0 SM |
| d_31_-16:0 SM | 734.6 [M+H]^+^ | **184.0 (28)** / 86.1 (76) | 12.7 | - |
| 16:0 SM | 703.6 [M+H]^+^ | **184.0 (8)** / 86.1 (76) | 12.8 | d_31_-16:0 SM |
| 18:0 SM | 731.6 [M+H]^+^ | **184.0 (28)** / 86.1 (76) | 14.7 | d_31_-16:0 SM |
| 20:0 SM | 759.6 [M+H]^+^ | **184.0 (28)** / 86.1 (78) | 17 | d_31_-16:0 SM |
| 22:0 SM | 787.7 [M+H]^+^ | **184.0 (28)** / 86.1 (78) | 19.4 | d_31_-16:0 SM |
| 24:0 SM | 815.7 [M+H]^+^ | **184.0 (28)** / 86.1 (80) | 22.7 | d_31_-16:0 SM |
| 24:1 SM | 813.7 [M+H]^+^ | 184.0 (8) / **86.1 (80)** | 19.5 | d_31_-16:0 SM |
| 17:0 Glucosyl-Cer | 714.6 [M+H]^+^ | **264.2 (44)** / 696.6 (12) | 13.2 | - |
| 16:0 Hexosyl-Cer | 700.6 [M+H]^+^ | **264.2 (40)** / 682.6 (12) | 12.5 | 17:0 Glucosyl-Cer |
| 24:1 Hexosyl-Cer | 810.7 [M+H]^+^ | **264.2 (40)** / 792.7 (16) | 18.8 | 17:0 Glucosyl-Cer |
| 17:0 Lactosyl-Cer | 876.6 [M+H]^+^ | **264.3 (52)** / 534.5 (24) | 12.6 | - |
| 16:0 Lactosyl-Cer | 862.6 [M+H]^+^ | **264.3 (48)** / 520.5 (20) | 12.1 | 17:0 Lactosyl-Cer |
| 24:1 Lactosyl-Cer | 972.7 [M+H]^+^ | **264.3 (56)** / 630.7 (28) | 17.8 | 17:0 Lactosyl-Cer |

^a^ Abbreviations: Cer, ceramide; dhCer, dihydroceramide; dhSM, dihydrosphingomyelin; dhSph, dihydrosphingosine; S1P, sphingosine-1-phosphate; SM, sphingomyelin; Sph, sphingosine.

^b^ Quantifiers are given in bold. Collision energies (in eV) are shown in parentheses.
