# Supplementary material for: Genome-wide identification of modulators of Chlamydia trachomatis parasitophorous vacuole stability highlights an important role for sphingolipid supply
Source: PLoS Biol. 2025 Aug 12;23(8):e3003297. doi: 10.1371/journal.pbio.3003297 (PMC12342332; doi:10.1371/journal.pbio.3003297)
Supplement: S1 Methods — Gene blocks used for the cloning of the GFP11-tagged constructs. Gene blocks used for the generation of vector pTL2-tetO-CTL0050-GFP11x4-FLAG-CTL0050 (pOmpA-GFP11int) and pTL2-tetO-IncB-GFP11x3-IncB-FLAG (pIncB-GFP11int). (DOCX) [file pbio.3003297.s011.docx]

**S1 Methods. Gene blocks used for the cloning of the GFP11-tagged constructs.** Gene blocks used for the generation of vector pTL2-tetO-CTL0050-GFP11x4-FLAG-CTL0050 (pOmpA-GFP11_int_) and pTL2-tetO-IncB-GFP11x3-IncB-FLAG (pIncB-GFP11_int_).

*Gene block (OmpA-GFP11_int_)*

GCGGCCGCATGAAAAAACTCTTGAAATCGGTATTAGTGTTTGCCGCTTTGAGTTCTGCTTCCTCCTTGCAAGCTCTGCCTGTGGGGAATCCTGCTGAACCAAGCCTTATGATCGACGGAATTCTATGGGAAGGTTTCGGCGGAGATCCTTGCGATCCTTGCACCACTTGGTGTGACGCTATCAGCATGCGTATGGGTTACTATGGTGACTTTGTTTTCGACCGTGTTTTGCAAACAGATGTGAATAAAGAATTCCAAATGGGTGCCAAGCCTACAACTGCTACAGGCAATGCTGCAGCTCCATCCACTTGTACAGCAAGAGAGAATCCTGCTTACGGCCGACATATGCAGGATGCTGAGATGTTTACAAATGCTGCTTACATGGCATTGAATATTTGGGATCGTTTTGATGTATTCTGTACATTAGGAGCCACCAGTGGATATCTTAAAGGAAATTCAGCATCTTTCAACTTAGTTGGGTTATTCGGAGATAATGAGAACCATGCTACAGTTTCAGATAGTAAGCTTGTACCAAATATGAGCTTAGATCAATCTGTTGTTGAGTTGTATACAGATACTACTTTTGCTTGGAGTGCTGGAGCTCGTGCAGCTTTGTGGGAATGTGGATGCGCGACTTTAGGCGCTTCTTTCCAATACGCTCAATCCAAGCCTAAAGTCGAAGAATTAAACGTTCTCTGTAACGCAGCTGAGTTTACTATCAATAAGCCTAAAGGATATGTAGGGCAAGAATTCCCTCTTGATCTTAAAGCAGGAACAGATGGTGTGACAGGAACTAAGGATGCCTCTATTGATTACCATGAATGGCAAGCAAGTTTAGCTCTCTCTTACAGACTGAATATGTTCACTCCCTACATTGGAGTTAAATGGTCTCGAGCAAGTTTTGATGCAGACACGATTCGTATTGCTCAGCCGAAGTCAGCTACAACTGTCTTTGATGTTACCACTCTGAACCCAACTATTGCTGGAGGTTCGGGACGTGACCACATGGTCCTTCATGAGTATGTAAATGCTGCTGGGATTACAGGTGGCTCTGGAGGTAGAGATCATATGGTTCTCCACGAATACGTTAACGCCGCAGGCATCACTGGCGGATCAGGTGGCAGGGATCACATGGTACTCCATGAATATGTGAACGCTGCTGGAATCACAGGCGGTAGCGGCGGTCGGGACCATATGGTCTTGCACGAATATGTCAATGCTGCCGGTATCACCATGGACTACAAGGATGACGACGATAAGTCAGCTGGCGATGTGAAAGCTAGCGCAGAGGGTCAGCTCGGAGATACCATGCAAATCGTTTCCTTGCAATTGAACAAGATGAAATCTAGAAAATCTTGCGGTATTGCAGTAGGAACAACTATTGTGGATGCAGACAAATACGCAGTTACAGTTGAGACTCGCTTGATCGATGAGAGAGCTGCTCACGTAAATGCACAATTCCGCTTCTAATAAGTCGAC

*Gene block (IncB-GFP11_int_)*

CGGCCGATGGTTCATTCTGTATACAATTCATTGGCTCCAGAAGGTTTTAGCCAAGTCTCTATTCAACCCAGTCAGATTCCAACCAGCAAAAAAGTAATGATTGCGATAATGACTCTTTTTGCACTCACAGCCATTGCAGCAATAGTCCTTTCCATCGTTACAGTTTGTGGAGGGTTTCCTTTTCTTCTTGCTGCACTTAACGGTTCGGGACGTGACCACATGGTCCTTCATGAGTATGTAAATGCTGCTGGGATTACAGGTGGCTCTGGAGGTAGAGATCATATGGTTCTCCACGAATACGTTAACGCCGCAGGCATCACTGGCGGATCAGGTGGCAGGGATCACATGGTACTCCATGAATATGTGAACGCTGCTGGAATCACAATGTCAACCGTAACTATTGGTGCATGCGTATCCTTGCCGATATTTACTTGCATAGCTACAACGTTATTACTTCTTTGTCTCCGTAATATCGAACTCCTAGCCAGACCGCAAGTATTGACCCTCTCCACTCAATTCAGCCCAACAAAACCTCAAGAAGACTACAAGGATGACGACGATAAGTAATAAGTCGAC

*Legend*

Restriction sites (NotI/EagI and SalI) Start codons Genes encoding OmpA (aa 1-326 and aa 327-394) or IncB (aa 1-65 and aa 66-115) GFP1-11 repeats Linker sequences FLAG tag Stop codons
