# Supplementary material for: Genome-wide identification of modulators of Chlamydia trachomatis parasitophorous vacuole stability highlights an important role for sphingolipid supply
Source: PLoS Biol. 2025 Aug 12;23(8):e3003297. doi: 10.1371/journal.pbio.3003297 (PMC12342332; doi:10.1371/journal.pbio.3003297)
Supplement: S1 Raw Images — Raw images of western blots shown in Figs 2E, S3B, and S6C. (PDF) [file pbio.3003297.s012.pdf]

S1 Raw Images
KDSR KO

Part of Fig 2E

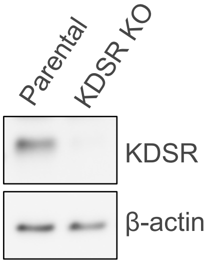

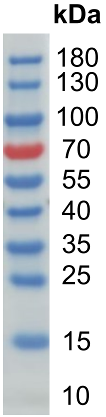

Original images

Chemiluminescence + marker

Chemiluminescence only

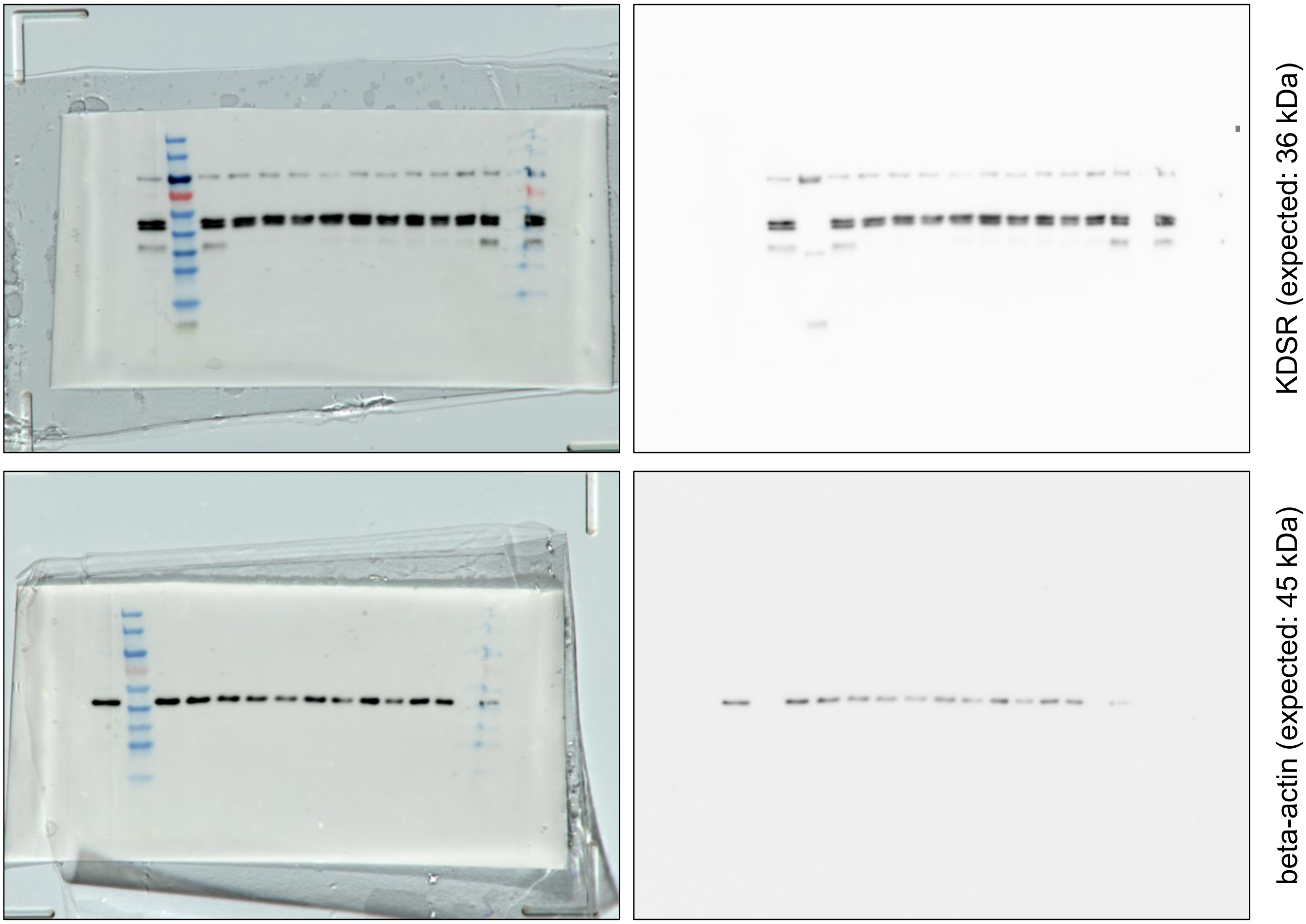

Only the first two lanes following the ladder are shown in Fig 2E. The other lanes show samples of other batches or clones of cells not used in this study.

S1 Raw Images
SPTLC1 KD

Part of Fig 2E

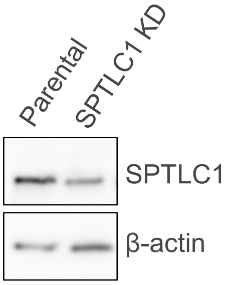

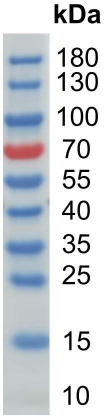

Original images

Chemiluminescence + marker

Chemiluminescence only

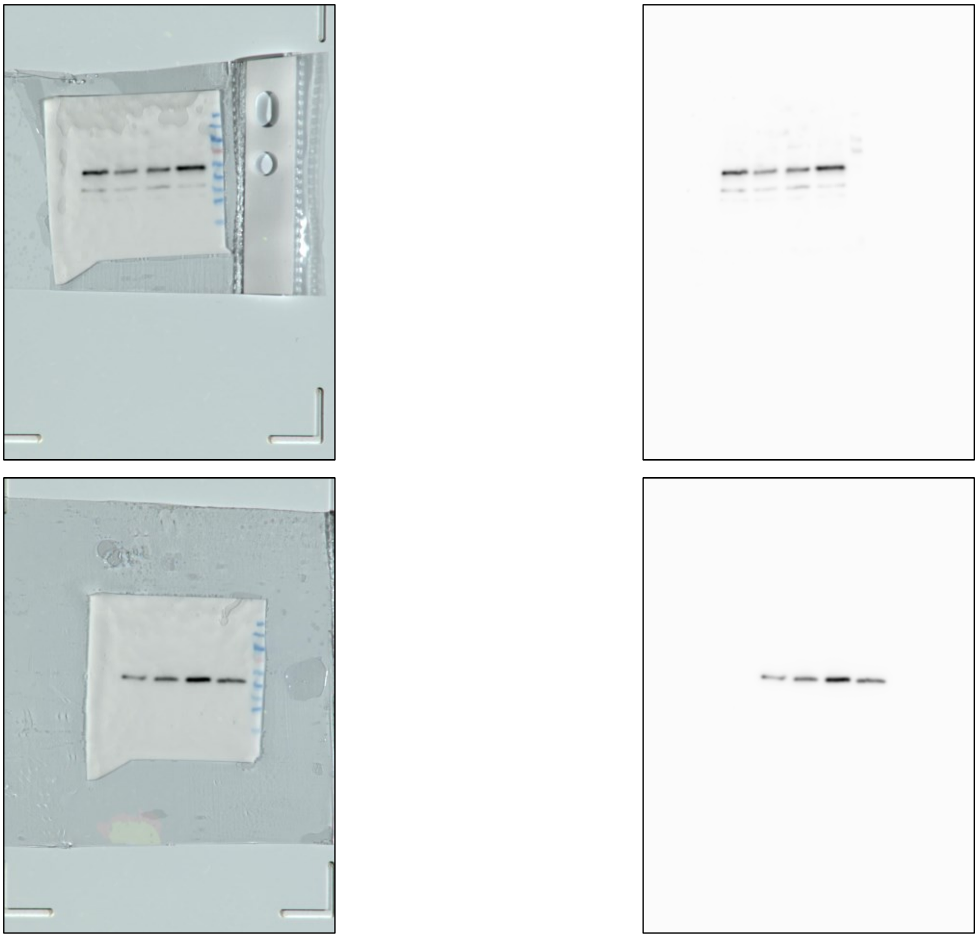

SPTLC1 (expected: 53 kDa)

beta-actin (expected: 45 kDa)

Only the first two lanes are shown in Fig 2E. The other lanes show samples of other batches or clones of cells not used in this study.

S1 Raw Images
COG3 KO

Part of S3B Fig

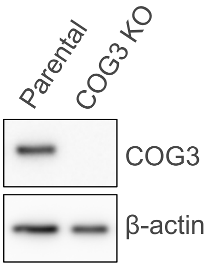

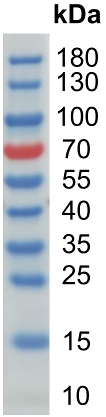

Original images

Chemiluminescence + marker | Chemiluminescence only

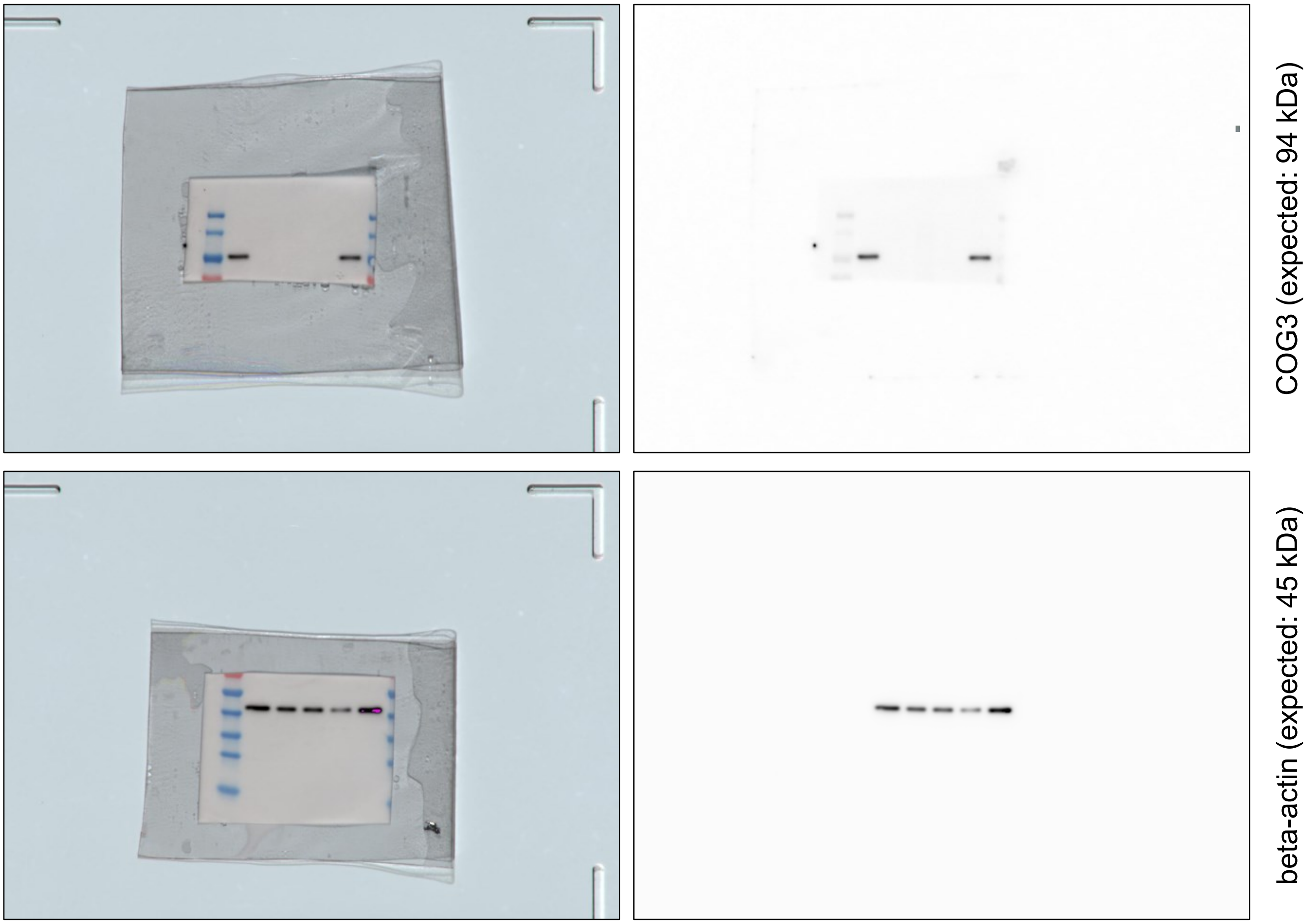

Only the first two lanes following the ladder are shown in S3B Fig. The other lanes show samples of other batches or clones of cells not used in this study.

S1 Raw Images
EXT1 KO

Part of S3B Fig

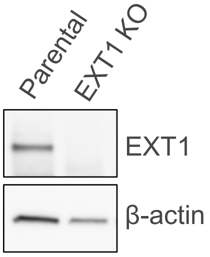

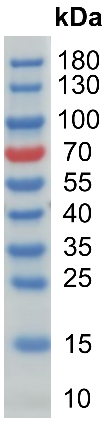

Original images

Chemiluminescence + marker

Chemiluminescence only

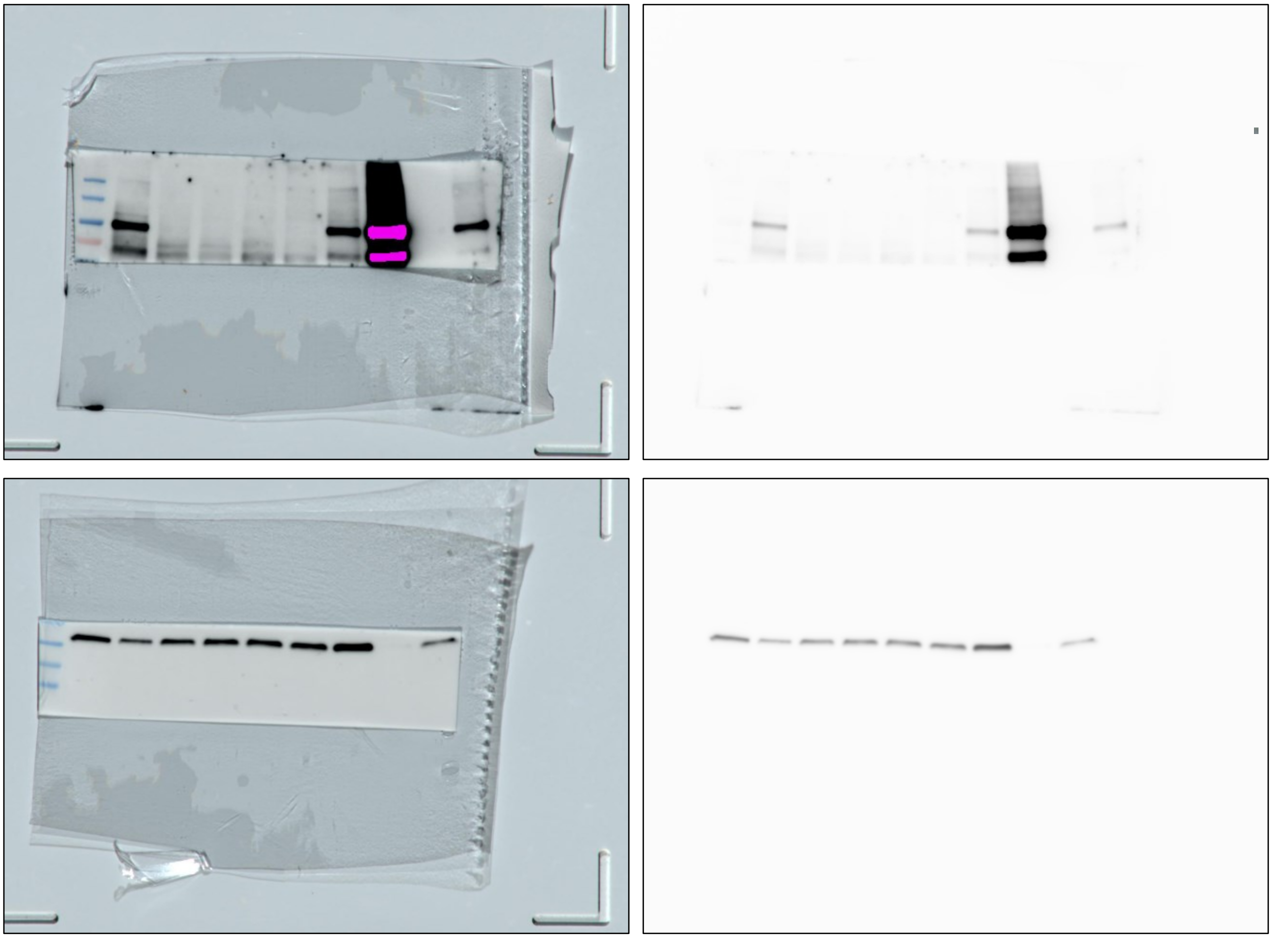

EXT1 (expected: 86 kDa)

beta-actin (expected: 45 kDa)

Only the first two lanes following the ladder are shown in S3B Fig. The other lanes show samples of other batches or clones of cells not used in this study.

S1 Raw Images
CERT KD

Part of S6C Fig

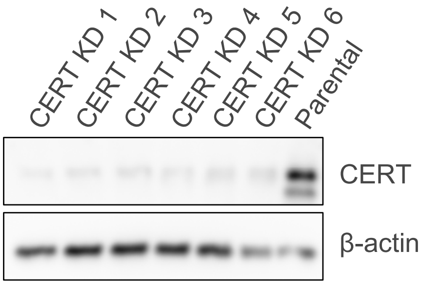

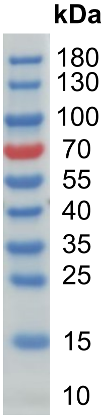

Original images

Chemiluminescence + marker

Chemiluminescence only

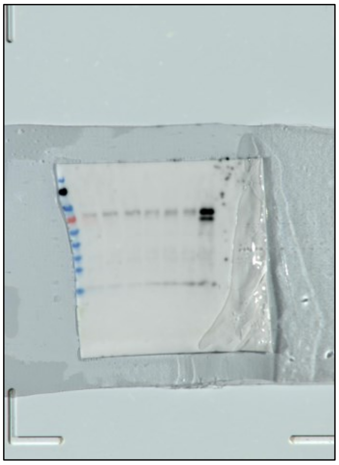

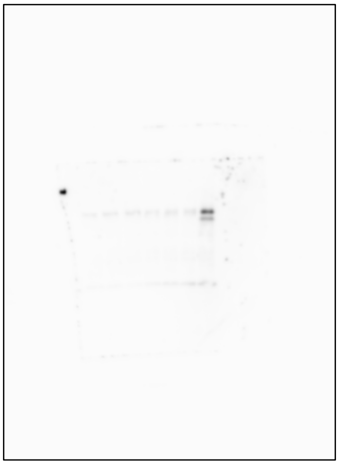

CERT (expected: 71 kDa)

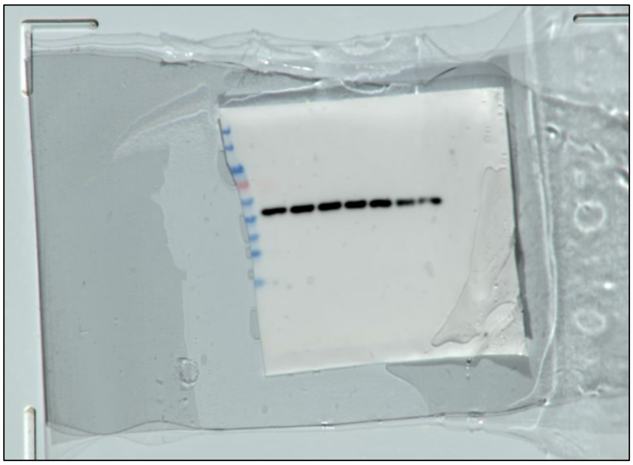

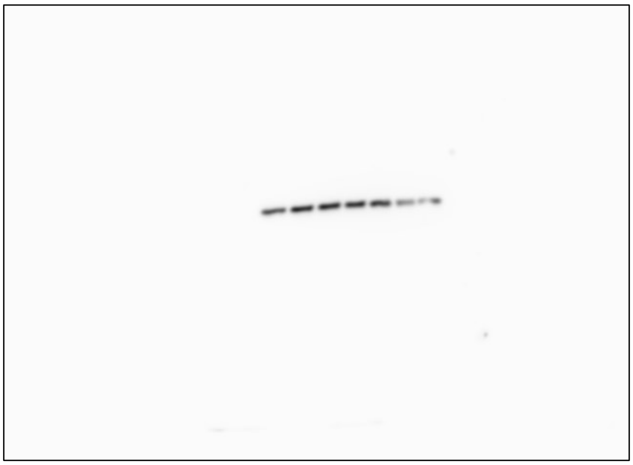

beta-actin (expected: 45 kDa)

All lanes following the ladder are shown in S6C Fig.
